# Drug‐induced shortening of the electromechanical window is an effective biomarker for in silico prediction of clinical risk of arrhythmias

**DOI:** 10.1111/bph.14786

**Published:** 2019-09-04

**Authors:** Elisa Passini, Cristian Trovato, Pierre Morissette, Frederick Sannajust, Alfonso Bueno‐Orovio, Blanca Rodriguez

**Affiliations:** ^1^ Department of Computer Science University of Oxford Oxford UK; ^2^ SALAR, Safety and Exploratory Pharmacology Department, Merck Research Laboratories Merck & Co., Inc. West Point PA USA

## Abstract

**Background and Purpose:**

Early identification of drug‐induced cardiac adverse events is key in drug development. Human‐based computer models are emerging as an effective approach, complementary to in vitro and animal models. Drug‐induced shortening of the electromechanical window has been associated with increased risk of arrhythmias. This study investigates the potential of a cellular surrogate for the electromechanical window (EMw) for prediction of pro‐arrhythmic cardiotoxicity, and its underlying ionic mechanisms, using human‐based computer models.

**Experimental Approach:**

In silico drug trials for 40 reference compounds were performed, testing up to 100‐fold the therapeutic concentrations (EFTPC_max_) and using a control population of human ventricular action potential (AP) models, optimised to capture pro‐arrhythmic ionic profiles. EMw was calculated for each model in the population as the difference between AP and Ca^2+^ transient durations at 90%. Drug‐induced changes in the EMw and occurrence of repolarisation abnormalities (RA) were quantified.

**Key Results:**

Drugs with clinical risk of Torsade de Pointes arrhythmias induced a concentration‐dependent EMw shortening, while safe drugs lead to increase or small change in EMw. Risk predictions based on EMw shortening achieved 90% accuracy at 10× EFTPC_max_, whereas RA‐based predictions required 100× EFTPC_max_ to reach the same accuracy. As it is dependent on Ca^2+^ transient, the EMw was also more sensitive than AP prolongation in distinguishing between pure hERG blockers and multichannel compounds also blocking the calcium current.

**Conclusion and Implications:**

The EMw is an effective biomarker for in silico predictions of drug‐induced clinical pro‐arrhythmic risk, particularly for compounds with multichannel blocking action.

**Abbreviations**
∆EMw: electromechanical window change by drug action
AP: action potential
APD_XX_: action potential duration at XX% of repolarisation
CTD_XX_: Ca^2+^ transient duration at XX% of the initial base value
DA: depolarisation abnormalities
dV/dt_MAX_: maximum upstroke velocity
EFTPC_max_: maximal effective therapeutic free concentration
EMw: electromechanical window
FN: false negative(s)
FP: false positive(s)
G_X_: I_X_ conductance
h: Hill coefficient
I_CaL_: L‐type Ca^2+^ current
I_K1_: inward rectifier K^+^ current
I_Kr_: rapid delayed rectifier K^+^ current
I_Ks_: slow delayed rectifier K^+^ current
I_Na_: fast Na^+^ current
I_NaK_: Na^+^–K^+^ pump current
I_NaL_: late Na^+^ current
I_NCX_: Na^+^–Ca^2+^ exchanger current
I_to_: transient outward K^+^ current
ORd: O'Hara‐Rudy dynamic human ventricular model
RA: repolarisation abnormalities
RMP: resting membrane potential
TdP: Torsade de Pointes
TN: true negative(s)
TP: true positive(s)
Tri_90‐40_: AP triangulation
V_peak_: peak voltage

**What is already known**

- Human in silico drug trials can predict clinical risk of drug‐induced arrhythmia with high accuracy
- Shortening of the electro‐mechanical window in vivo is associated with pro‐arrhythmia, but controversy exists

**What this study adds**

- In silico, the electro‐mechanical window increases predictive accuracy of drug‐induced arrhythmias at clinically relevant doses
- The electro‐mechanical window is more effective than alternative biomarkers as it reflects intracellular calcium changes.

**What is the clinical significance**

- In silico drug trials can contribute to the development of safer and more efficient medicines
- Human‐based computer models can identify subpopulations of patients vulnerable to drug‐induced cardiac arrhythmias

## 1 INTRODUCTION

Early prediction of drug‐induced cardiotoxicity is key during drug development and still remains a major challenge (Laverty et al., 2011; Stevens & Baker, 2009). Animal models are widely used for preclinical in vitro, ex vivo, and in vivo studies, but safety findings do not always translate to humans (Berridge et al., 2013), and predictions of clinical risk of arrhythmias for large sets of compounds still show limited accuracy (Lawrence, Bridgland‐Taylor, Pollard, Hammond, & Valentin, 2006; Valentin et al., 2009). The current ICH S7B/E14 guidelines (2005b, 2005a) focus on hERG channel block and QTc prolongation, as surrogate markers of pro‐arrhythmia. Although this paradigm has been effective in preventing new pro‐arrhythmic drugs from entering the market, it has important limitations and may have led to stopping the development of potentially valuable therapeutics (Sager, Gintant, Turner, Pettit, & Stockbridge, 2014). This suggests that new effective strategies and biomarkers, for a more comprehensive prediction of drug‐induced cardiotoxicity in patients, are needed in the preclinical stages of drug development. In silico human‐based methodologies are becoming increasingly established in pharmacology as a potential alternative to animal experiments in the early phases of drug development, using a variety of approaches (Abbasi, Small, Patel, Jamei, & Polak, 2017; Britton, Abi‐Gerges, et al., 2017; Chang et al., 2017; Dutta et al., 2017; Krogh‐Madsen, Jacobson, Ortega, & Christini, 2017; Lancaster & Sobie, 2016; Li et al., 2017, 2019; Paci, Passini, Severi, Hyttinen, & Rodriguez, 2017; Passini et al., 2017; Rodriguez et al., 2016). We recently demonstrated that human in silico drug trials can achieve high accuracy (close to 90%) in pro‐arrhythmic cardiotoxicity prediction for more than 60 reference compounds (Passini et al., 2017). The occurrence of repolarisation abnormalities (RA), which are mechanistically linked with arrhythmias, was shown to be a sensitive biomarker to predict clinical drug‐induced arrhythmic risk using populations of human ventricular in silico action potential (AP) models. However, the best prediction accuracy was achieved for drug concentrations up to 100‐fold the maximal effective free therapeutic concentration (EFTPC_max_). While investigating the effect of potential overdoses is important in drug safety assessment, testing drug concentrations much larger than the EFTPC_max_ might lead to false positives (Krogh‐Madsen et al., 2017).

The electromechanical window (EMw), defined as the difference between the duration of electrical and mechanical systole, has been suggested as a promising biomarker to predict clinical risk of Torsade de Pointes (TdP) arrhythmia in several preclinical animal models (Guns, Johnson, Van Op den Bosch, Weltens, & Lissens, 2012; Guns, Johnson, Weltens, & Lissens, 2012; Morissette et al., 2016; van der Linde et al., 2010). However, for dofetilide, Stams et al. (2014) showed that, in the in vivo atrioventricular block canine model, the EMw solely reflects changes in QT prolongation and therefore lacks specificity for prediction of drug‐induced TdP.

The aim of this study was to investigate the potential of a cellular surrogate for the EMw as a biomarker for predictions of clinical drug‐induced arrhythmic risk using human in silico trials for 40 reference compounds. We hypothesised that the EMw was a more sensitive biomarker of arrhythmic risk than AP prolongation and RA, particularly for multichannel block involving both potassium and calcium currents, given its dependency with calcium transient duration.

## 2 METHODS

### 2.1 Design of the control population of models

A control population of human ventricular AP models was constructed and optimised, blindly to the drug trials results. The population was constructed using the O'Hara‐Rudy dynamic (ORd) model (O'Hara, Virág, Varró, & Rudy, 2011) as baseline and the experimentally calibrated population of models methodology (Britton et al., 2013; Muszkiewicz et al., 2016; Passini et al., 2017). The nine main ionic conductances were randomly varied: fast and late Na^+^ (G_Na_ and G_NaL_ respectively), transient outward K^+^ (G_to_), rapid and slow delayed rectifier K^+^ (G_Kr_ and G_Ks_), inward rectifier K^+^ (G_K1_), Na^+^–Ca^2+^ exchanger (G_NCX_), Na^+^–K^+^ pump (G_NaK_), and the L‐type Ca^2+^ (G_CaL_). The ranges of variation of each conductance are shown in Table 1, and they were optimised based on results in Passini et al. (2017), to maximise the number of models accepted in the population while at the same time minimising the population size. In brief, models with severe G_Na_, G_Kr,_ G_NaK_, or G_K1_ down‐expression often fail to produce physiological APs, while models with low repolarisation reserve (increased G_CaL_, G_NaL_, and G_NCX_, reduced G_NaK_, G_Kr_, and G_Ks_) are more prone to develop drug‐induced RA. Using this process, an initial population of 150 models was produced.

**Table 1:** Variability ranges for each of the nine parameters in the control population, expressed as percentage of the baseline model values

Model parameter	Variability range
G_Na_	[30–200]%
G_NaL_	[100–200]%
G_to_	[0–200]%
G_Kr_	[45–100]%
G_Ks_	[0–100]%
G_K1_	[30–200]%
G_NCX_	[100–200]%
G_NaK_	[30–100]%
G_CaL_	[100–200]%

Abbreviations: G_Na_/G_NaL_, fast/late Na^+^ current conductance; G_to_, transient outward K^+^ current conductance; G_Kr_/G_Ks_, rapid/slow delayed rectifier K^+^ current conductance; G_K1_, inward rectifier K^+^ current conductance; G_NCX_, Na^+^–Ca^2+^ exchanger; G_NaK_, Na^+^–K^+^; G_CaL_, L‐type Ca^2+^ current conductance.

This initial population was paced at 1 Hz for 500 beats (to allow the models to reach steady state), and the last AP trace for each model was used to compute a set of seven AP and two Ca^2+^ transient biomarkers: AP duration at 40%, 50%, and 90% of repolarisation (APD_40,_ APD_50_, and APD_90_); AP triangulation, defined as the difference between APD_90_ and APD_40_ (Tri_90‐40_); maximum upstroke velocity (dV/dt_MAX_); peak voltage (V_peak_); resting membrane potential (RMP); and Ca^2+^ transient duration at 50% and 90% of repolarisation (CTD_50_ and CTD_90_). Both AP and Ca^2+^^ ^durations were computed starting from the instant of maximum dV/dt_MAX_.The population was then filtered based on experimental AP recordings (Britton, Bueno‐Orovio, Virág, Varró, & Rodriguez, 2017; O'Hara et al., 2011), and Ca^2+^ transient recordings (Coppini et al., 2013; Passini et al., 2016) from undiseased human hearts (*N* = 37 hearts, *n* = 62 cells for AP, *N* = 8 hearts, *n* = 25 cells for Ca^2+^ transient). Out of the initial 150 AP models, 107 displayed biomarkers within the experimental ranges (Table 2) and were then used for the in silico drug trials. The refinement of ionic conductance ranges significantly enhanced model acceptance in the final population to >70% compared to ~40% in previous studies (Passini et al., 2017). It is worth noting that variability in the experimental data may be caused in part by experimental interventions, such as the isolation procedure, in addition to the original variability in ion channel density. However, with the population approach, we assume that different sources of variability can be globally modelled by varying ion channel conductances.

**Table 2:** Experimental AP (Britton, Bueno‐Orovio, et al., 2017; O'Hara et al., 2011; Passini et al., 2017) and Ca^2+^ transient (Coppini et al., 2013; Passini et al., 2016) biomarker ranges used to calibrate the control population of human ventricular AP in silico models

AP biomarker	Min value	Max value
APD_40_	85 ms	320 ms
APD_50_	110 ms	350 ms
APD_90_	180 ms	440 ms
Tri_90‐40_	50 ms	150 ms
dV/dt_MAX_	100 V/s	1000 V/s
V_peak_	10 mV	55 mV
RMP	−95 mV	−80 mV
CTD_50_	120 ms	420 ms
CTD_90_	220 ms	785 ms

Abbreviations: APD_XX_, AP duration at XX% of repolarisation; CTD_XX_, Ca^2+^ transient duration at XX% of decay; dV/dt_MAX_, maximum upstroke velocity; RMP, resting membrane potential; Tri_90‐40_, AP triangulation, defined as the difference between APD_90_ and APD_40_; V_peak_, peak voltage.

All the simulations presented in this study were conducted using Virtual Assay (v.2.4.800 © 2014 Oxford University Innovation Ltd. Oxford, UK), a user‐friendly software package based on C++, and specifically designed for in silico drug assays in populations of computer models. The verification of Virtual Assay results against additional software packages has already been established (Passini et al., 2017). Further analysis of the results was performed in Matlab (Mathworks Inc. Natwick, MA, USA, RRID:SCR_001622).

### 2.2 Human in silico drug trials

In silico drug trials were performed in the population of 107 human AP models for 40 reference compounds. Drug effects were simulated using a simple pore‐block model (Brennan, Fink, & Rodriguez, 2009), with IC_50_ and Hill coefficient (h) acquired internally in whole‐cell automated patch clamp configuration, for four ion channels: fast Na^+^ current (I_Na_), rapid/slow delayed rectified K^+^ current (I_Kr_/I_Ks_), and L‐type Ca^2+^ current (I_CaL_). Details on ion channel inhibition protocols are included in the Supporting Information. Additional data on drug/ion channel interactions have been integrated from publications for relevant compounds, as detailed in Supporting Information. Multiple concentrations were investigated for each compound, from 1 to 100‐fold the maximal effective free therapeutic concentration (EFTPC_max_). The full list of reference compounds, together with IC_50_/h, EFTPC_max_, and the TdP risk category considered for this study, is included in Table S1.

Each drug was assigned to a TdP risk category, based on the classification by CredibleMeds® (Woosley & Romer, 1999), available on www.crediblemeds.org (Accessed November 30, 2018): 1 (known risk), drugs which prolong the QT interval and are clearly associated with a known risk of TdP, even when taken as recommended; 2 (possible risk), drugs which can cause QT prolongation but currently lack evidence for a risk of TdP risk when taken as recommended; 3 (conditional risk), drugs associated with TdP but only under certain circumstances of their use, for example, excessive dose, in patients with conditions such as hypokalaemia, or when taken with interacting drugs; NC (not classified), the drug was reviewed by CredibleMeds® but the evidence available did not result in a decision for it to be placed in any risk categories. Of the 40 considered compounds, 22 are categorised as known risk and eight as potential/conditional risk, for a total of 30 drugs associated with TdP risk. Verapamil, mexiletine, and diltiazem (categorised as NC) and the remaining seven compounds (not listed in CredibleMeds®) are considered as safe (no TdP risk) for the purpose of this study. The choice of reference compounds evaluated in this study was determined by the ion channel information available internally. Out of the 40 reference compounds, 22 were also investigated earlier by Passini et al., (2017), but using ion channel information from a different source.

**Figure 1:**
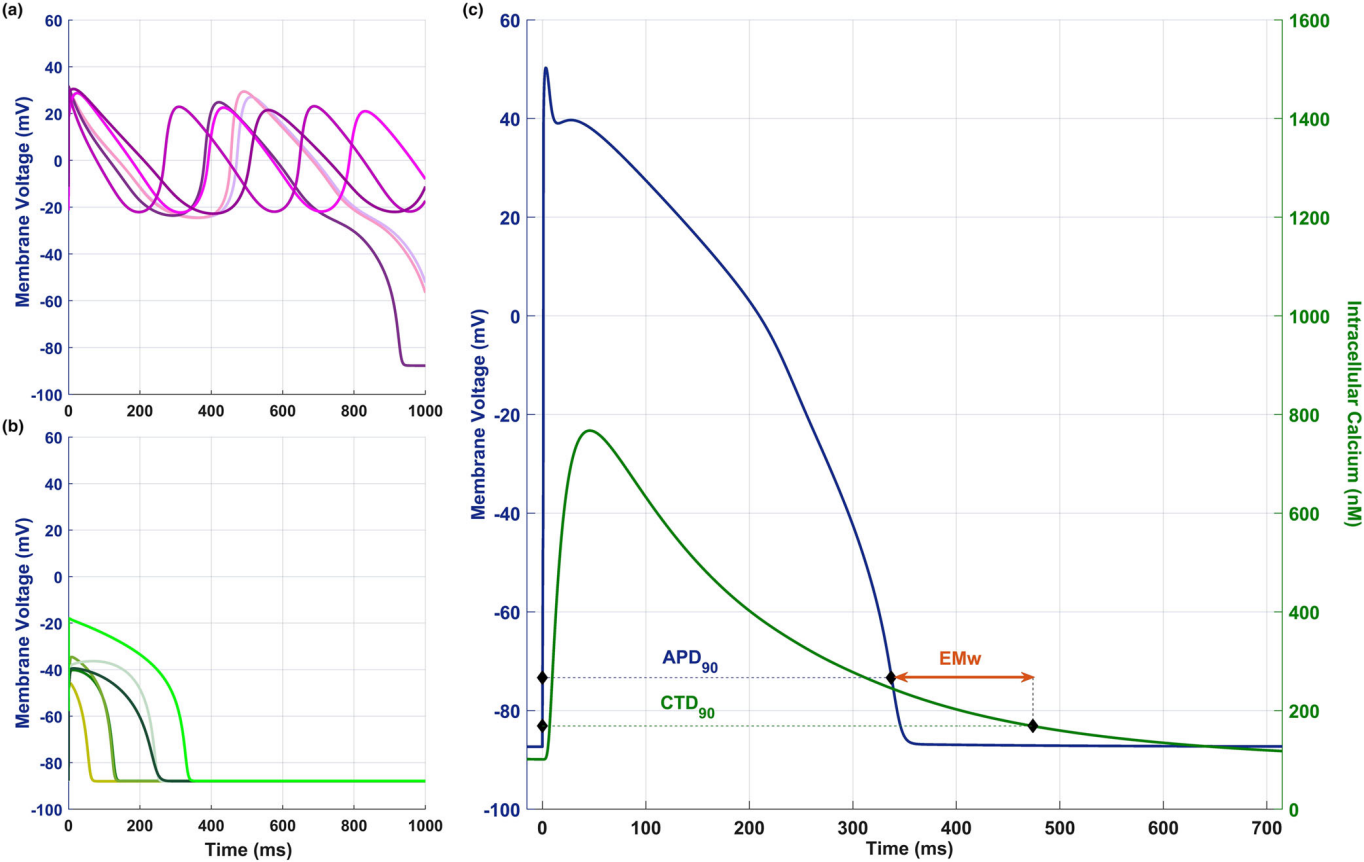
Drug‐induced AP phenotypes and APD_90_, CTD_90_ and EMw biomarkers. (a,b) Representative AP traces illustrating repolarisation abnormalities (a) and depolarisation abnormalities (b). (c) In silico EMw, defined here as the difference between CTD_90_ and APD_90_

For the human in silico drug trials, all 107 models in the experimentally calibrated control population were paced at 1 Hz for 150 beats, for each drug concentration. The last AP and Ca^2+^ transient traces of each simulation were compared with the corresponding control (150 beats at 1 Hz without drug). All AP traces were automatically checked for repolarisation and depolarisation abnormalities (RA and DA respectively), as in Passini et al. (2017). Representative traces of these drug‐induced AP phenotypes are shown in Figure 1a,b. For models not displaying abnormalities, APD_90_ and CTD_90_ were computed as described above, together with the EMw, defined as the difference between CTD_90_ and APD_90_, as shown in Figure 1c.

### 2.3 TdP risk prediction and TdP score

In silico results were used to predict the TdP risk of each drug, based on two different criteria: (a) occurrence of drug‐induced RA—a drug is classified as risky if it provokes RA in at least one model of the in silico population, as in Passini et al. (2017); (b) drug‐induced changes in the EMw (∆EMw)—a drug is classified as risky if it shortens the EMw more than 10% (∆EMw < −10%; Morissette et al., 2016). To quantify ∆EMw in the in silico population, the median of the drug‐induced percentage changes of each model compared with the corresponding control was considered. The two above criteria will be hereafter referred to as “RA only” and “RA + ∆EMw.” It is worth noting that, following drug application, EMw distributions in the population vary up to four orders of magnitude: Therefore, EMw data are shown in a logarithmic scale, using the log‐modulus transformation (L(x) = sign(x) * log(|x| + 1)), which spreads out the smaller data while preserving their sign (John & Draper, 1980).

By comparing in silico results against the TdP risk categories, drug‐risk predictions were divided into true positives (TP, drug with TdP risk classified as risky); true negatives (TN, drug with no TdP risk classified as safe); FP (drug with no TdP risk, classified as risky); and false negatives (FN, drugs with TdP risk, classified as safe). Prediction performances were evaluated based on sensitivity = TP/(TP + FN); specificity = TN/(TN + FP); accuracy = (TN + TP)/(TN + TP + FN + FP); positive predictive value = TP/(TP + FP); and negative predictive value = TN/(TN + FN).

The scoring system based on RA occurrence proposed in Passini et al. (2017), in order to integrate the results obtained at different concentrations, was extended to include the information provided by the EMw, according to the following formulas:

$$\mathrm{new}\;\mathrm{TdP}\;\text{score}=\frac{\sum_{i}\left[w_{i}*\left(n{\mathrm{RA}}_{i}+n{\mathrm{EMw}}_{i}\right)\right]}{n_{\mathrm{tot}}*\sum_{i}\left(w_{i}\right)}\;\text{original}\;\mathrm{TdP}\;\text{score}=\frac{\sum_{i}\left(w_{i}*n{\mathrm{RA}}_{i}\right)}{n_{\mathrm{tot}}*\sum_{i}\left(w_{i}\right)}\;\text{from Passini}\;\mathrm{et}\;\mathrm{al}.\left(2017\right),$$

where *nRA*_*i*_ is the number of models showing RA at the tested concentration *i* (*C*_*i*_), *nEMw*_*i*_ is the number of models for which *∆EMw*_*i*_ <  − 10%, *w*_*i*_ = EFTPC_max_/*C*_*i*_ is the weigth inversely related to the tested concentration *C*_*i*_, and *n*_*tot*_ is the total number of models in the population. For each tested concentration *C*_*i*_, the score considers the fraction of models showing drug‐induced RA (*nRA*_*i*_/*n*_*tot*_) or shortening of the EMw (*nEMw*_*i*_/*n*_*tot*_) beyond threshold. All contributions are multiplied for a weight inversely related to their testing concentration (e.g., 1/30 for *nRA*_*i*_+*nEMw*_*i*_ observed at 30× EFTPC_max_) and added together for metric normalisation. Therefore, the final TdP score varies between 0 and 1, where 0 corresponds to a drug that does not provoke RA nor EMw shortening beyond threshold in any of the models in the population, while 1 corresponds to a drug that shows either RA or EMw shortening beyond threshold in 100% of the models, at all tested concentrations. By using the proposed score, RA and EMw shortening are naturally considered more severe when occurring at lower concentrations and/or affecting a higher fraction of the population of models.

### 2.4 Ion channel block sensitivity analysis

To investigate the difference in drug‐induced TdP risk predictions across different biomarkers (RA, EMw, and APD_90_), we performed a sensitivity analysis of multichannel drug blocks for the three ion channels which are known to play a major role in RA generation (Passini et al., 2016): I_Kr_, I_CaL_, and I_NaL_. Simulations were run for all the combinations of five different current block percentages (0%, 25%, 50%, 75%, and 100%), for a total of 5^3^ = 125 simulations. For each simulation, changes in the EMw and APD_90_ compared to control were considered, as well as the fraction of models displaying either RA, EMw shortening beyond threshold (<−10%, as described above) or APD_90_ prolongation beyond threshold (>6%, as defined in Passini et al., 2017). The data and statistical analysis comply with the recommendations of the British Journal of Pharmacology on experimental design and analysis in pharmacology.

### 2.5 Nomenclature of targets and ligands

Key protein targets and ligands in this article are hyperlinked to corresponding entries in http://www.guidetopharmacology.org, the common portal for data from the IUPHAR/BPS Guide to PHARMACOLOGY (Harding *et al*., 2018), and are permanently archived in the Concise Guide to PHARMACOLOGY 2017/18 (Alexander, Kelly et al., 2017; Alexander, Striessnig et al., 2017).

## 3 RESULTS

### An optimised human in silico control population of models

**Figure 2:**
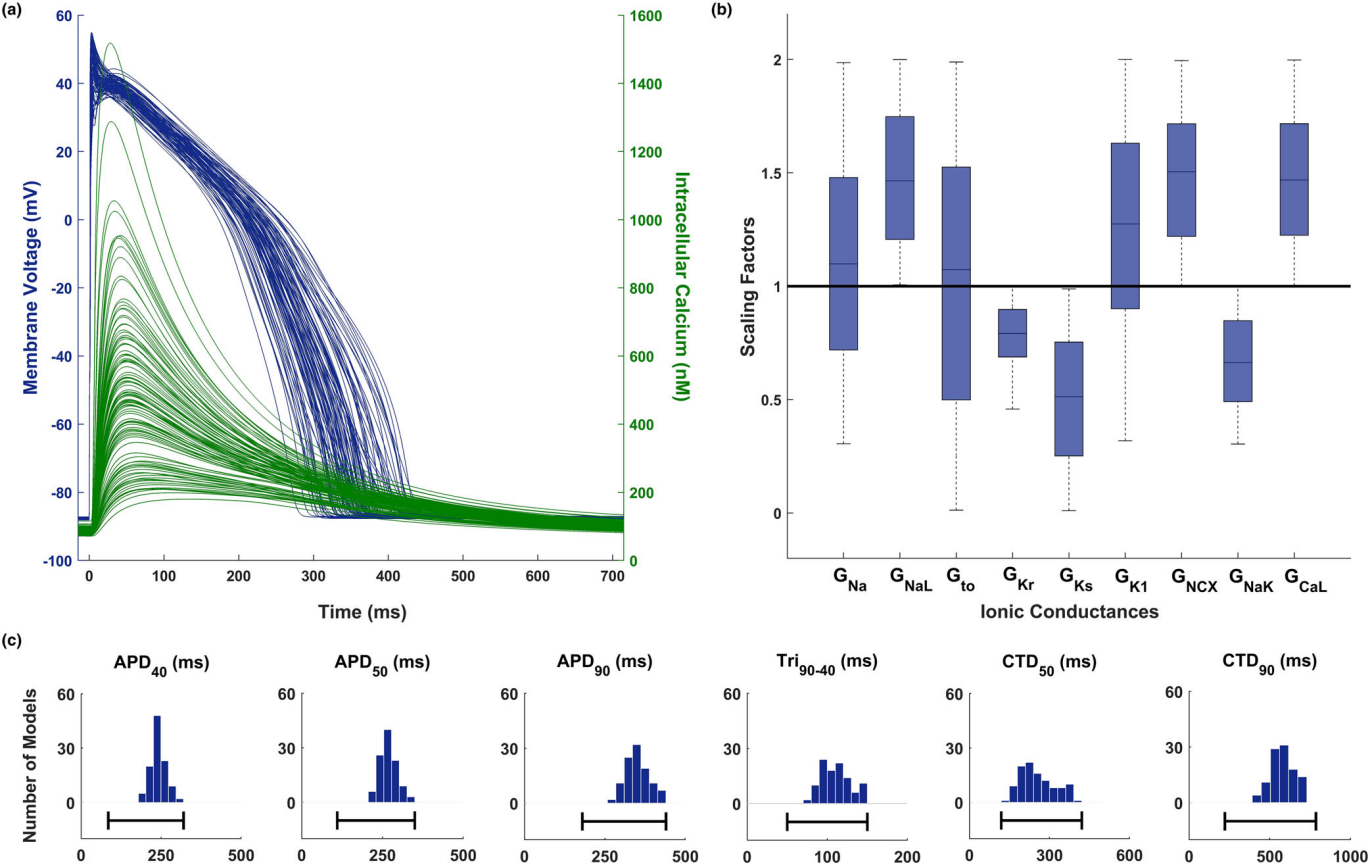
Optimised population of human ventricular models. (a) AP and Ca^2+^ transient traces of the 107 models in the calibrated population (blue and green respectively). (b) Ionic profile of the calibrated population, shown as scaling factors with respect to the baseline model. Central mark corresponds to median, box limits are the 25^th^ and 75^th^ percentiles, and whiskers extend to the most extreme data points. (c) AP and Ca^2+^ transient biomarker distributions for the calibrated population (blue histograms) and experimental ranges (black lines): AP duration at 40%, 50%, and 90% of repolarisation (APD_40,_ APD_50,_ and APD_90_); AP triangulation, as difference between APD_90_ and APD_40_ (Tri_90‐40_); Ca^2+^ transient duration at 50% and 90% of decay (CTD_50_ and CTD_90_)

Figure 2 shows an illustrative summary of the human control population of 107 models, including AP and Ca^2+^ transient traces (Figure 2a, blue and green respectively); ionic profile boxplots (Figure 2b), reflecting the ranges defined in Table 1; AP and Ca^2+^ transient biomarker distributions (Figure 2c) and the corresponding human experimental ranges defined in Table 2 (black lines). All the 107 models show a healthy‐looking AP phenotype, with all the AP and Ca^2+^ transient biomarkers within the experimental ranges. They also have, by design, a weak repolarisation reserve (Varshneya, Devenyi, & Sobie, 2018), that is, the main repolarising currents are down‐regulated, while the main inward currents are up‐regulated. It is worth noting that, mainly due to I_Kr_ down‐regulation, the AP biomarkers more representative of the repolarisation phase (APD_40,_ APD_50,_ APD_90_, and Tri_90‐40_) are distributed in the upper part of the experimental ranges.

### 3.2 Risky drugs induce shortening of the in silico EMw

**Figure 3:**
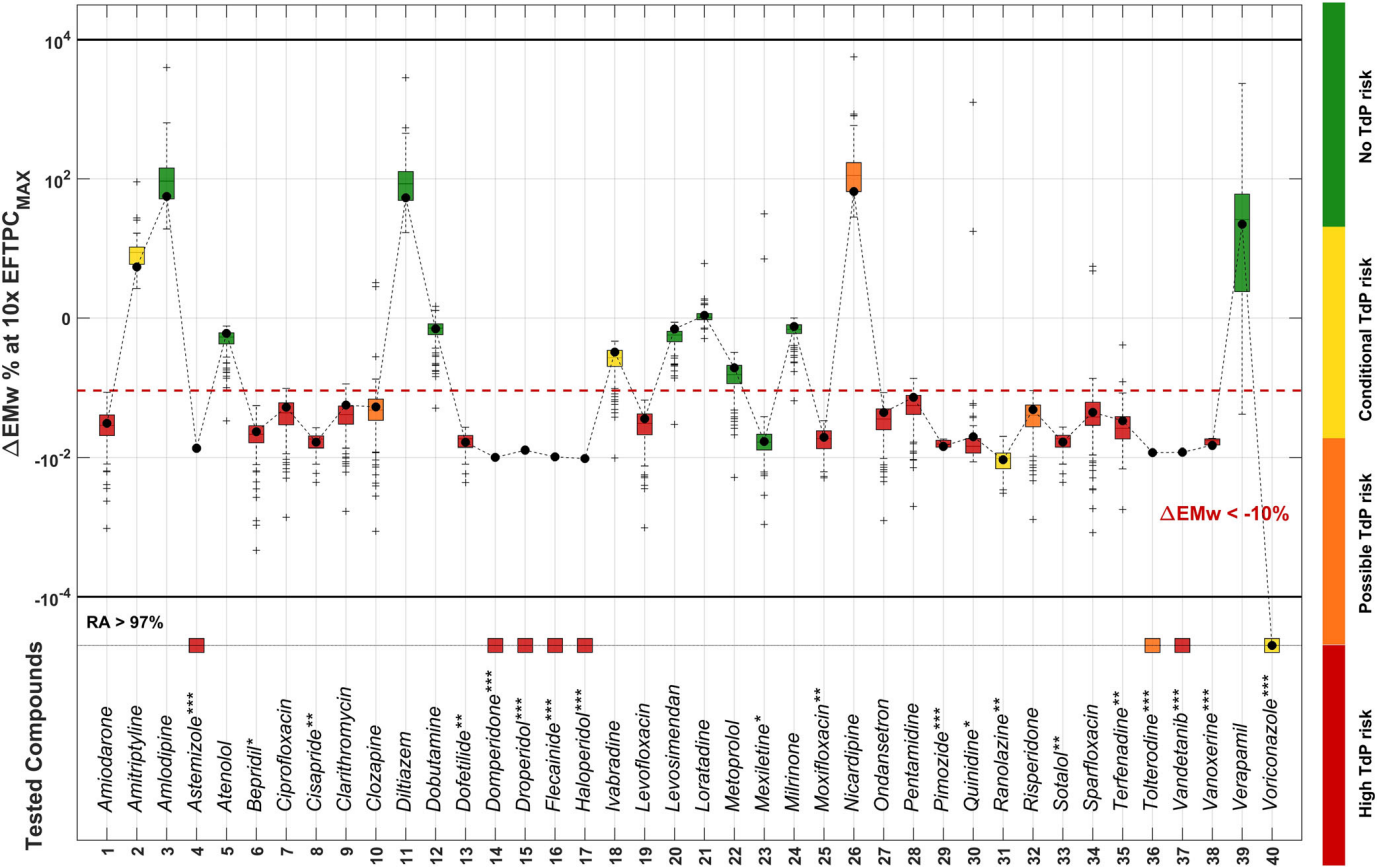
Drug‐induced changes in EMw (∆EMw) in the in silico population for 40 compounds at 10× EFTPC_max_. Data are shown using the log‐modulus transformation, as described in Section 2. ∆EMw shortening threshold (−10%) is marked as a dashed red line. Colour scale (green to red) corresponds to TdP risk category: no TdP risk (green), conditional (yellow), possible (orange), and known (red) TdP risk. Drug‐induced RA occurrence is marked by one or more asterisks, based on the fraction of models displaying RA: * (>10%), ** (>30%), *** (>50%). Boxplots are not shown for drugs exhibiting more than 97% RA (three models or less for which to compute the EMw), and these are depicted on the dotted line at the bottom of the figure. Boxplots description as in Figure 2. Result obtained by using the single ORd model are shown as black dots, connected by a dashed black line

Figure 3 shows the ∆EMw distributions computed using the in human in silico model population for the 40 reference compounds, tested at 10× EFTPC_max_. Drugs inducing EMw shortening (∆EMw < −10%) are classified as risky (boxplots with median below the red dashed line), while drugs prolonging the EMw or inducing below‐threshold ∆EMw changes are considered safe (boxplots with median above the red dashed line). Many of the drugs shortening the EMw also induce RA in the population of models (marked with asterisks). All drugs with known TdP risk (red boxplots) are correctly predicted as risky using the in silico EMw. All safe drugs (green boxplots) are correctly classified as safe, except for Mexiletine. Only three drugs with possible/conditional TdP risk (orange/yellow boxplots) are categorised as safe: amitriptyline, ivabradine, nicardipine. Drug‐induced ∆EMw increase in magnitude with the tested concentrations (Figures S1–S2). For testing concentrations of 30× and 100× EFTPC_max_, some compounds categorised as safe (i.e., ivabradine, metoprolol, and verapamil) can induce EMw shortening beyond threshold (Figure S2).

It is worth noting that, for all drugs, 75% of the models are either above or below the 10% EMw threshold, including the baseline ORd model (black dots, connected by a dashed black line in Figure 3). Even though predictions based on a single model would yield the same prediction accuracy as the population, for many drugs, such as ciprofloxacin, clarithromycin, clozapine, levofloxacin, ondansetron, pentamidine, and risperidone), the drug‐induced EMw changes for the baseline model are very close to the threshold. In those cases, the population results provide additional evidence on the confidence and robustness of the simulations, thus reinforcing the results.

### 3.3 Shortening of the EMw improves TdP risk prediction at low concentrations

**Figure 4:**
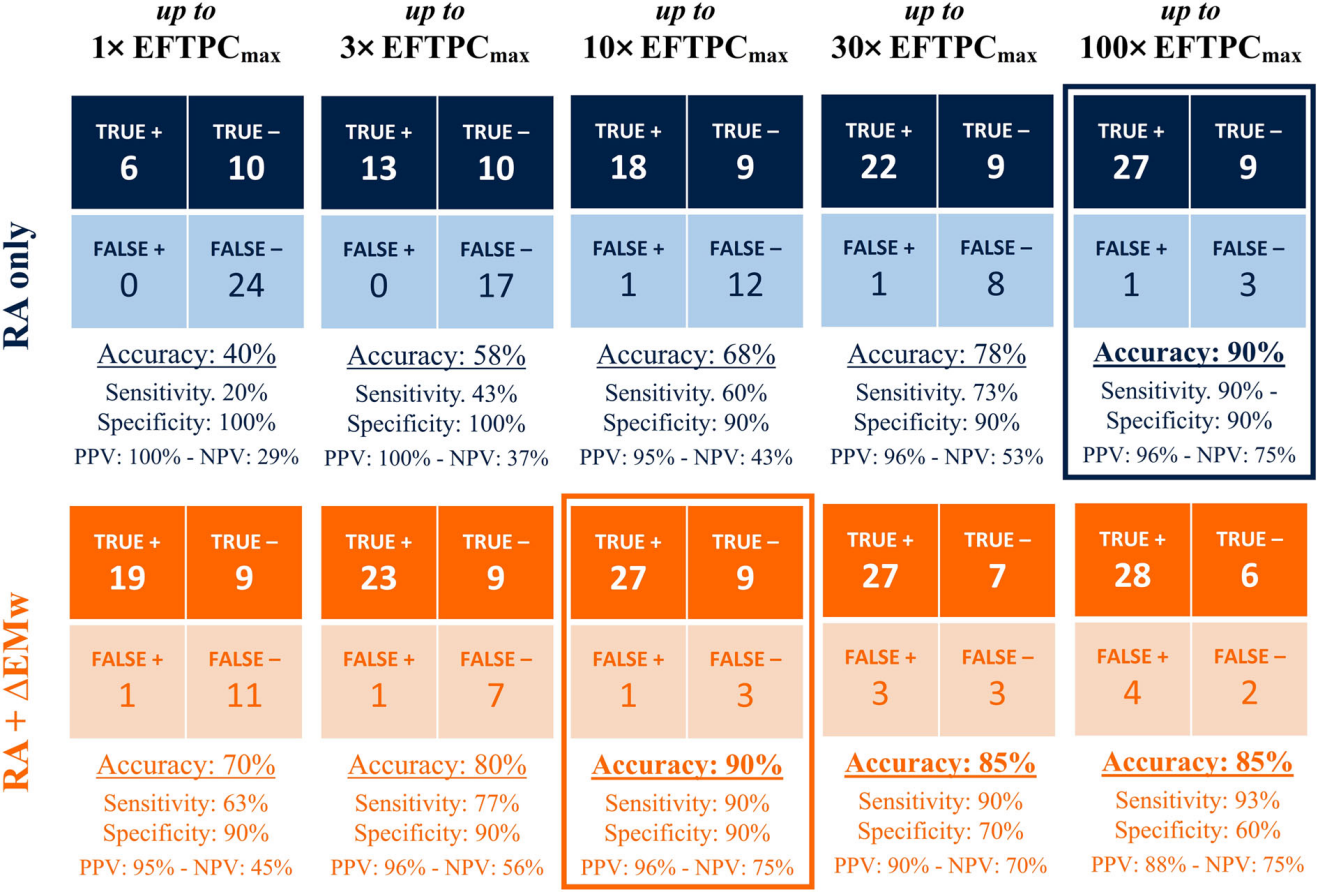
Clinical TdP risk predictions for 40 reference compounds, at five concentrations (multiples of EFTPC_max_) in the human in silico population of models, using RA occurrence only (top line, in blue), or using RA + ∆EMw (bottom line, in orange). Predictions with accuracy >80% are shown in bold. Predictions with highest accuracy are highlighted with a rectangle

Figure 4 shows a summary of the in silico predictions of clinical TdP risk for the 40 reference compounds at all tested concentrations, based on the two criteria described in Section 2: RA only (top line, in blue); RA + ∆EMw (bottom line, in orange). The two different criteria reach the same maximum accuracy of 90% (highlighted with a rectangle), with 27/30 risky drugs classified as risky, 9/10 safe drugs classified as safe, and misclassified drugs as in Figure 3 (FN: amitriptyline, ivabradine, nicardipine; FP: mexiletine). However, the concentration at which the 90% accuracy is reached is substantially lower for predictions based on EMw shortening (10× vs. 100× EFTPC_max_). For both criteria, sensitivity always increases with the tested concentrations, whereas specificity decreases at very high drug concentrations for RA + ∆EMw.

**Figure 5:**
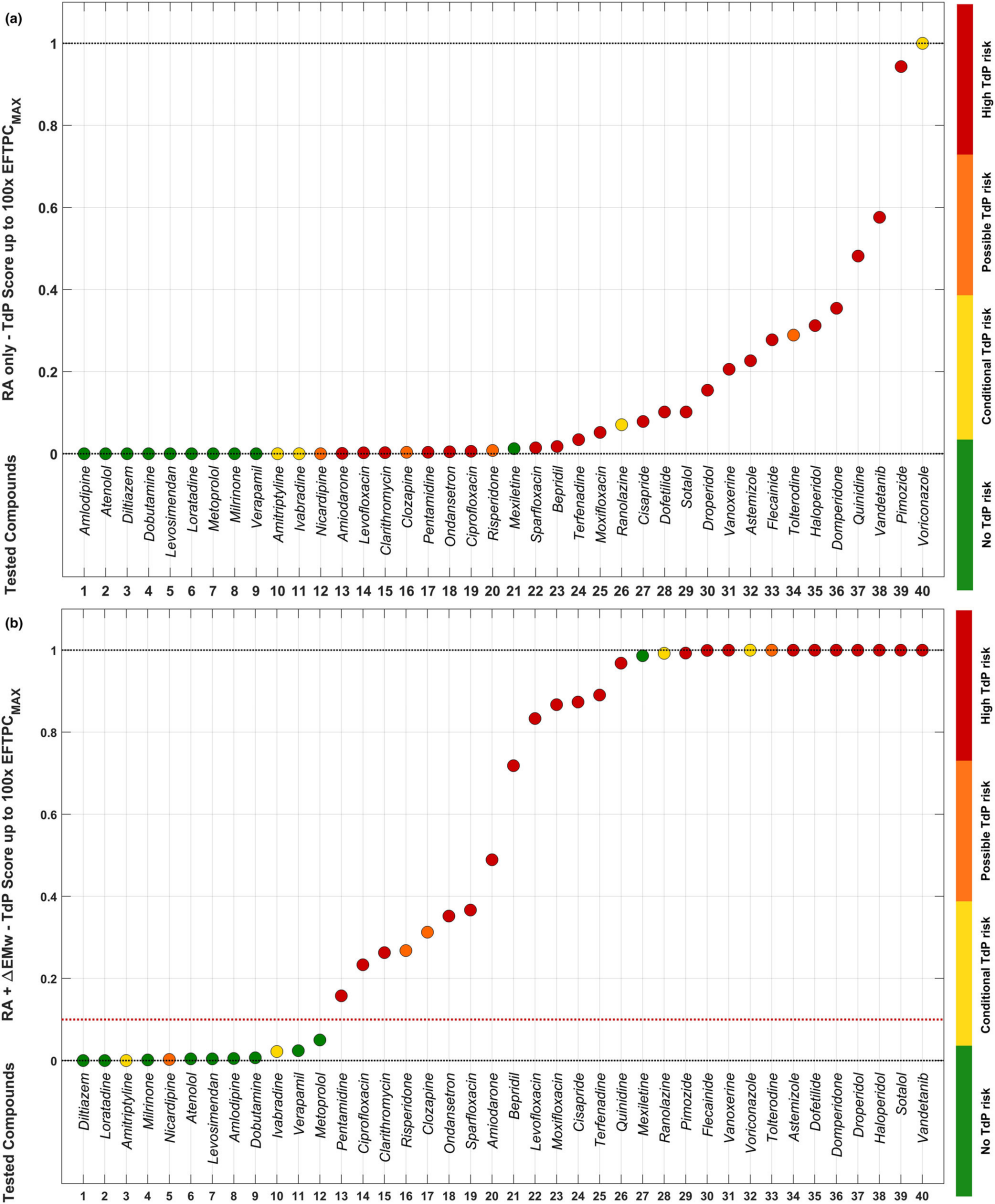
TdP scores, computed for the 40 reference compounds considering RA only (a) and RA + ∆EMw (b). Compounds with a low TdP score are predicted to be safe, while compounds with a high TdP score are predicted to be risky. Drug TdP risk categories as in Figure 3, from green to red

Figure 5 illustrates how the additional information provided by drug‐induced EMw shortening in the TdP score improves the separation between safe and risky drugs in the TdP score plot, with respect to using RA only (Figure 5a vs. 5b). Results shown in Figure 5a are also depicted in Figure S3 with a logarithmic scale, required to appreciate separation for low TdP values. The four drugs that are misclassified according to the TdP score plot correspond to the FN/FP in Figure 3.

### 3.4 The EMw is strongly correlated with Ca^2+^ transient biomarkers but not to APD_90_

**Figure 6:**
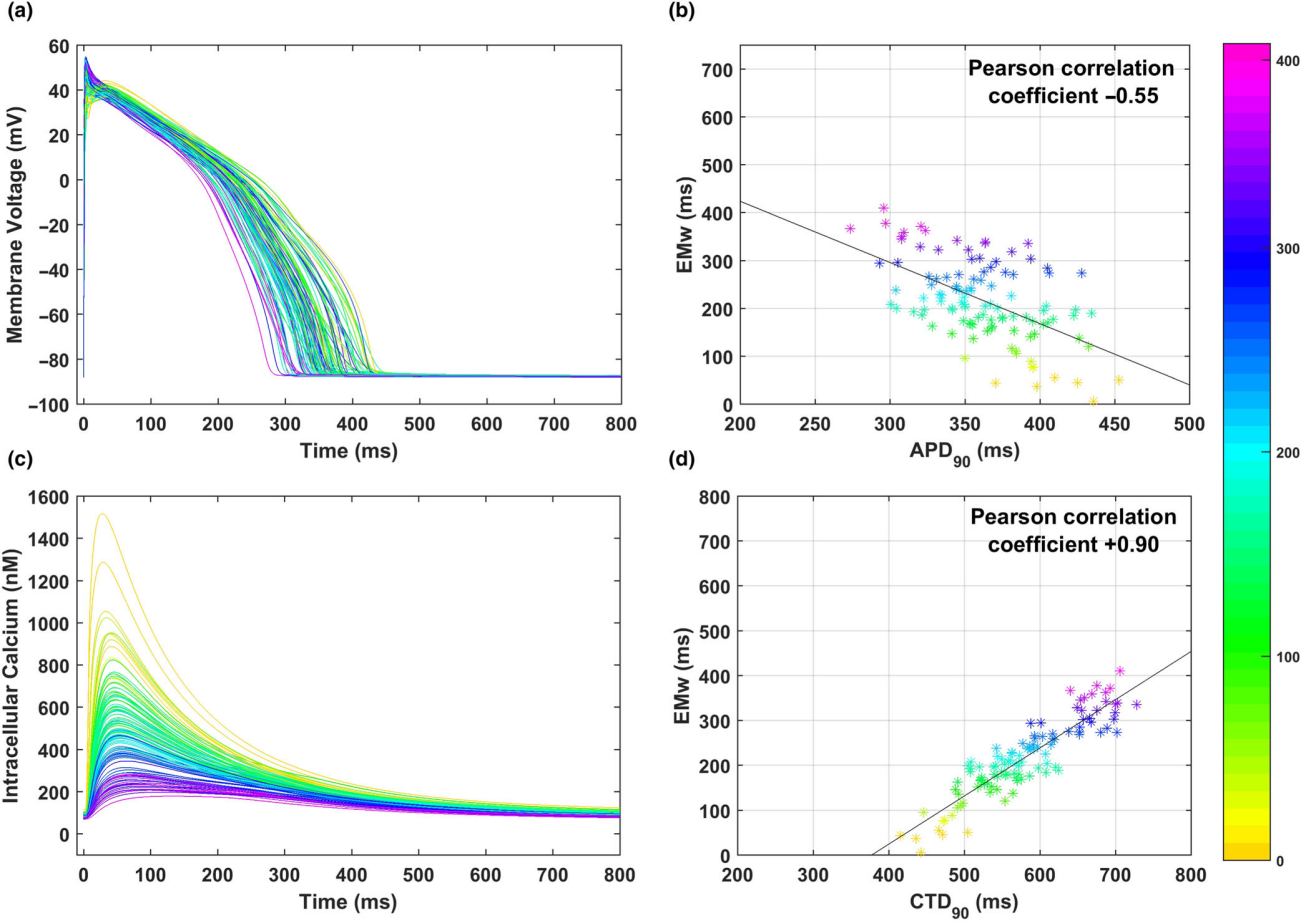
Relationship between APD_90_, CTD_90_, and EMw, in the 107 human ventricular AP models in control. All data are colour coded (bar on the right) based on the magnitude of the corresponding EMw, from 0 ms (yellow) to 410 ms (dark pink). (a) AP traces for the control population. (b) EMw versus APD_90_, and their regression line. (c) Ca^2+^ transient traces for the control population. (d) EMw versus CTD_90_, and their regression line

Figure 6 illustrates the relationship between EMw and APD_90_ or CTD_90_ in panels 6a and 6b, and 6c and 6d respectively. All traces and dots are colour‐coded based on the corresponding EMw magnitude. Figure 6a,b shows a weak correlation between EMw and APD_90_, whereas a strong correlation is observed between EMw and CTD_90_ (Figure 6c,d), with Pearson correlation coefficients equal to −0.55 and +0.90 for APD_90_ and CTD_90_ respectively. Models in the population with the same APD_90_ can have substantially different EMw, whereas a low Ca^2+^ transient peak always translates into a long CTD_90_ (the 90% threshold of Ca^2+^ transient decay is closer to 0), which in turn contributes to a large EMw.

This is particularly relevant when considering multichannel drug effects affecting ion channels with different block potencies. As an example, Figure S4 shows three different combinations of ion channel block, all inducing the same APD_90_ prolongation (+44% with respect to control), but with very different outcomes in terms of CTD_90_, and in turn EMw: I_Kr_ block; I_Kr_ + I_CaL_ block; and I_Kr_ + I_NaL_ block. When blocking I_Kr_ only, or I_Kr_ + I_NaL_, the APD_90_ is prolonged, but there is almost no effect in CTD_90_, thus resulting in an overall shortening of the EMw. On the contrary, when I_CaL_ block is also present, the APD_90_ prolongation is accompanied by a CTD_90_ prolongation, and therefore, no change is observed in the EMw. It is worth noting that both I_CaL_ and I_NaL_ blocks counteract the APD_90_ prolongation induced by I_Kr_ block: This is why a larger I_Kr_ block is needed to obtain the same APD_90_ prolongation when also including a 50% I_CaL_ or I_NaL_ blocks.

### 3.5 The EMw is more sensitive to Ca^2+^ current block than APD_90_

**Figure 7:**
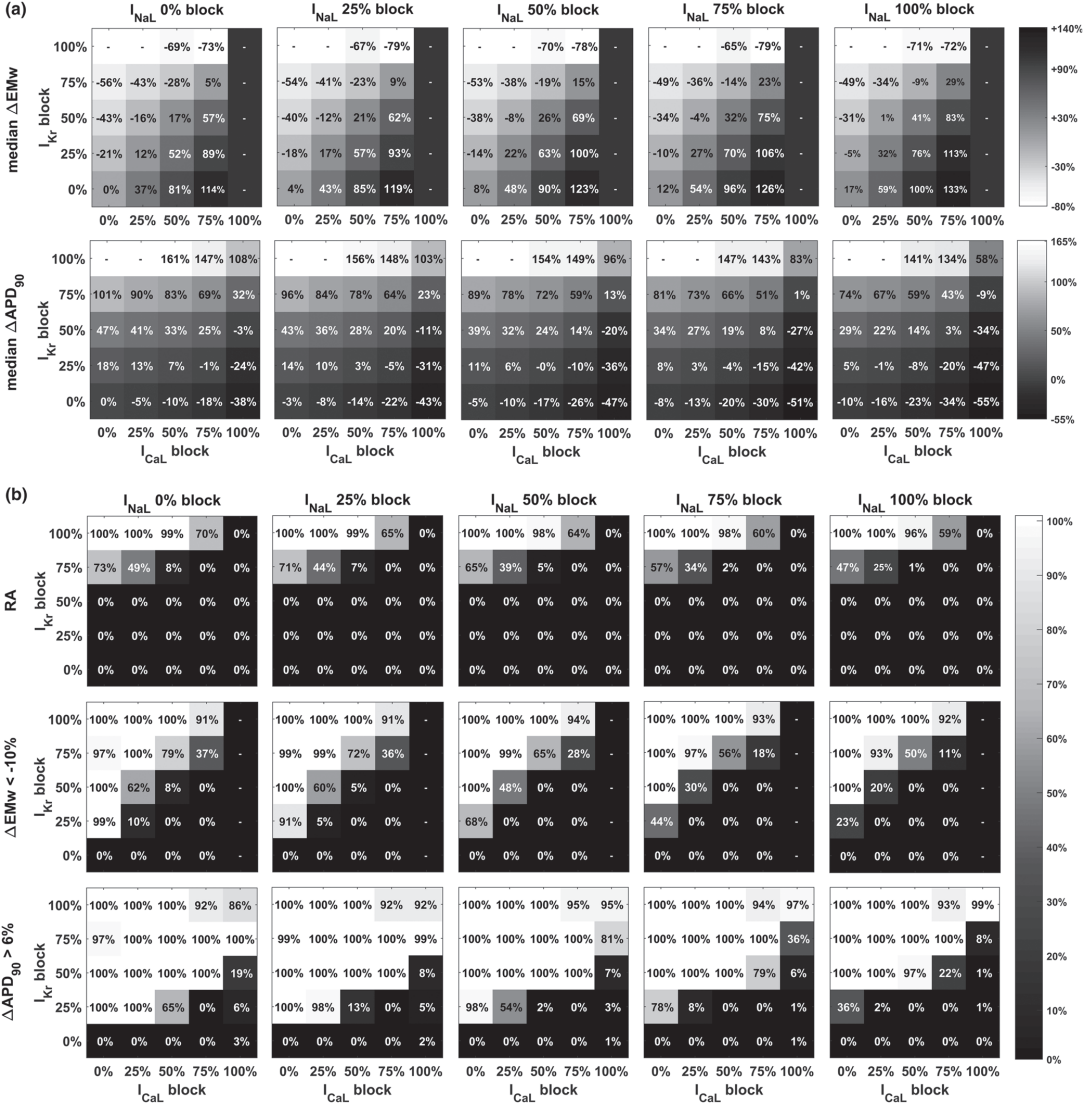
(a) Effect of multiple ion channel blocks (I_Kr_, I_CaL_, and I_NaL_) on APD_90_ (bottom row) and EMw (top row). Each column corresponds to a fixed percentage of I_NaL_ block (from 0% to 100%, left to right), and each single plot summarises the results for the 25 combinations of I_Kr_ and I_CaL_ blocks (y and x axis respectively). Values shown are the median changes in the 107 human models. “‐“ indicates that value could not be obtained due to occurrence of RA or lack of Ca^2+^ transient (e.g., total I_CaL_ block). Opposite colour scales were used for EMw and APD_90_, from white to black for EMw and from black to white for APD_90_. Since drug‐induced TdP risk is associated with EMw shortening and APD_90_ prolongation, for both biomarkers, white is therefore associated with high risk. (b) Effect of multiple ion channel blocks on the fraction of models displaying RA occurrence (top row), EMw shortening beyond threshold (middle row), and APD_90_ prolongation beyond threshold (bottom row). Numerical values are all in the range (0–100)%, coloured from black to white, and higher values correspond to block combinations predicted at higher risk for that specific biomarker

Figure 7 illustrates the results of investigations into the factors determining differences in drug‐induced TdP risk predictions for the different biomarkers (RA, EMw, and APD_90_) for combinations of I_Kr_, I_CaL_, and I_NaL_ blocks, from 0% to 100%.

Figure 7a shows the effect of the combinations of considered ion channel blocks on EMw (top row) and APD_90_ (bottom row). Each column corresponds to a fixed percentage of I_NaL_ block (from 0% to 100%, left to right), and each single plot summarises the results for the 25 combinations of I_Kr_ and I_CaL_ blocks (y and x axis respectively). The EMw shortens with the percentage of I_Kr_ block (from bottom to top, in each plot) and increases with the percentage of I_CaL_ block (from left to right, in each plot). When combining both blocks at the same time, the two opposite effects compensate each other, with I_CaL_ block playing the major role (e.g., 50% I_Kr_ and I_CaL_ blocks induce a ∆EMw = +17%). Shortening of the EMw is therefore observed only in the top‐left corner of each plot. APD_90_ increases with I_Kr_ block and decreases with I_CaL_ block. However, in this case, I_Kr_ has the predominant effect (e.g., 50% I_Kr_ and I_CaL_ blocks induce a ∆APD_90_ = +33%). I_NaL_ block has a positive effect on both biomarkers, contributing to counteract EMw shortening and APD_90_ prolongation caused by I_Kr_ block.

Figure 7b quantifies risk predictions for each of the scenarios explored in the sensitivity analysis of Figure 7a, with numerical values indicating in this case the fraction of models displaying: RA occurrence (top row), EMw shortening beyond threshold (middle row), and APD_90_ prolongation beyond threshold (bottom row). Numerical values are therefore all in the range (0–100)%, coloured from black to white, and higher values correspond to block combinations predicted at higher risk for those specific biomarkers. No RA occur when I_Kr_ is blocked less than 75%, in agreement with Britton, Bueno‐Orovio, et al., (2017). However, the fractions of models displaying RA is overall higher in this study, for example, 25% I_CaL_ block, 75% I_Kr_ block: 49% versus 1% of models displaying RA. This is due to the novel design of the population, with ionic conductances defined to maximise the likelihood of RA.

RA and EMw show a similar pattern: The fraction of models displaying RA or EMw shortening beyond threshold increases with I_Kr_ block and decreases with I_CaL_ block. The block scenarios at higher risk are distributed in the top‐left corner of each plot. However, changes in the EMw occur at lower percentage of I_Kr_ block, making it a more sensitive biomarker for low testing concentrations. On the contrary, APD_90_ prolongation is less sensitive to I_CaL_ block, compared to I_Kr_ block. Therefore, all the scenarios where I_Kr_ is blocked more than 25% would be considered as risky based on this biomarker only. This would lead to a misclassification of safe compounds which affect both I_CaL_ and I_Kr_, for example, verapamil, which at 3× EFTPCmax blocks I_Kr_ ~25% and I_CaL_ ~40% (based on IC_50_/h in Table S1). I_NaL_ block contributes to decreasing the fraction of models involved for each scenario, by contrasting APD_90_ prolongation. To better illustrate the effect of I_NaL_ block, Figure S5 shows the results of the sensitivity analysis with I_CaL_ and I_NaL_ block swapped.

## 4 DISCUSSION AND CONCLUSIONS

Human in silico drug trials were conducted for 40 reference compounds with multichannel blocking actions, to evaluate how drug‐induced EMw shortening can potentially improve predictions of clinical pro‐arrhythmic risk. A population of human ventricular AP models was optimised to capture pro‐arrhythmic ionic profiles, blindly to the drug trials results.

The main findings of this simulation study are the following:

1. Predictions of clinical TdP risk based on in silico EMw shortening have a high accuracy (90% for 40 reference compounds) at low tested concentrations (up to 10× EFTPC_max_). Predictions based on RA occurrence reach the same accuracy, but only when testing up to 100× EFTPC_max_. Reducing the maximum tested concentration is particularly important to minimise the risk of FP. It also reduces the simulation time required for each drug. The updated version of the TdP score, integrating information about both RA and EMw, also provides a better separation of safe/risky drugs compared to RA only (Figure 5).
2. EMw shortening is an effective biomarker for TdP risk prediction because it is more sensitive to I_CaL_ block compared to APD_90_. Indeed, taking into account drug‐induced changes in the EMw allows discrimination between pure I_Kr_ blockers, prolonging APD_90_ but with very little effect on Ca^2+^ transient, and those exhibiting also I_CaL_ block, where cardiac adverse events are less likely to occur.
3. The novel design for population of models, where ionic current conductances are sampled to maximise the models accepted during calibration as well as drug‐induced RA is successful for optimising populations for in silico drug trials. An accuracy comparable to our previous study (90% vs. 89% in Passini et al., 2017) was achieved by using about one tenth of the models, thus considerably reducing computing time (from 45 to 5 min for each compound at each concentration).

It is important to highlight that we correctly classified as risky all drugs with known TdP risk. When considering only drugs not listed in CredibleMeds (safe for the purpose of this study) and drugs with known TdP risk, our predictions show 100% accuracy, with 22 TP and eight TN. The results of this study are also fully consistent with previous in silico results, obtained with a similar methodology (Passini et al., 2017). Even if the two studies are based on different IC_50_/h data sets, the classification for the 22 compounds in common is equivalent: 18 TP, three TN, and one FP.

Compounds misclassified as FN (nicardipine, ivabradine, and amitriptyline) all belong to potential/conditional risk categories, which are usually associated with overdoses or interactions with other drugs, and often controversial. Two of them (nicardipine and ivabradine) have actually been considered as negative controls in previous studies (Champéroux et al., 2005; Morissette et al., 2016). By considering these two drugs as safe, our prediction accuracy would reach 95% (Sensitivity 96%, Specificity 92%). The last FN, mitriptyline, affects both I_Kr_ and I_CaL_ currents: The resulting safety profile is given by the balance between these two blocks, the former leading to QT prolongation and potentially TdP and the latter contributing to QT shortening and suppression of RA. Our input data could minimise amitriptyline risk by underestimating its effect on hERG trafficking (Dennis, Nassal, Deschenes, Thomas, & Ficker, 2011) and overestimating I_CaL_ IC_50_, for which controversial values have been reported (Crumb, Vicente, Johannesen, & Strauss, 2016; Lancaster & Sobie, 2016; Mirams et al., 2011; Zahradnı et al., 2008).

The only FP in this study is mexiletine, also misclassified in Passini et al. (2017). Mexiletine is a multichannel blocker, affecting mainly fast and late I_Na_, as well as I_Kr_. Based on a literature review, we can speculate that the main reason for the misclassification relies in an overestimation of I_Kr_ block when using a simple pore drug block model. Mexiletine binds preferably to the open state of the hERG channel (Gualdani et al., 2015), and predictions could potentially improve by using a dynamic hERG channel drug block model, as the one recently proposed by the FDA (Dutta et al., 2017; Li et al., 2017). A more detailed discussion on the FN/FP drugs is included in the Supporting Information.

Compared to the classification shown in Passini et al. (2017), based on RA occurrence alone, the inclusion of the EMw has the main advantage of decreasing the need to test very high concentrations (Krogh‐Madsen et al., 2017), while still considering a wide concentration range, which allows exploration of the drug effect induced by potential overdoses, as well as the EFTPCmax variability across patients. Testing EFTPCmax multiples is also the methodology proposed by within the Comprehensive in vitro Proarrhythmia Assay (CiPA) initiative (Li et al., 2019; Sager et al., 2014). Predictions based on EMw shortening achieved the same accuracy than those based on RA occurrence only, but at concentrations much closer to therapeutic doses (10× vs. 100× EFTPCmax). Reducing the range of tested concentrations also leads to a reduction of the simulation times required for each drug and, most importantly, limits the risk of inducing FP. While sensitivity increases with the tested concentration, specificity based on RA + ∆EMw tends to decrease at high concentrations, when even safe compounds can cause a large EMw shortening (Figure S3). FBs at high concentrations are less likely to occur for RA: A small degree of I_CaL_ block is sufficient to inhibit drug‐induced RA, even without counteracting APD_90_ prolongation and EMw shortening (Figure S6). The inclusion of the EMw also improves the separation between safe and risky drugs in the TdP score, originally presented in Passini et al. (2017). When considering the fraction of models displaying RA + ∆EMw, the TdP score of risky drugs increases due to the EMw contribution, allowing for a clearer risk classification. It is worth noting that all models displaying RA at a specific concentration also show EMw shortening at a lower one (Figure S7).

Even if the shortening of the EMw has been presented as an effective biomarker of pro‐arrhythmia in several experimental studies (Guns, Johnson, Van Op den Bosch, et al., 2012; Guns, Johnson, Weltens, & Lissens, 2012; Morissette et al., 2016; van der Linde et al., 2010), some controversy on its added value compared to APD_90_ prolongation still exists (Stams et al., 2014). Our sensitivity analysis, exploring 125 multiple combinations of I_Kr_, I_CaL_, and I_NaL_ blocks, showed how the EMw is more sensitive to drug‐induced changes in Ca^2+^ transient, and it is therefore able to distinguish between drugs which purely affect I_Kr_ and those with a multichannel action also modifying Ca^2+^ dynamics. As shown in Figure 7, risk predictions based on APD_90_ prolongation and EMw shortening are only in agreement when I_CaL_ block is equal to 0%, showing progressive disagreement when I_CaL_ block increases. Our results therefore explain why, in the in vivo canine atrioventricular block (CAVB) model under the effect of dofetilide “the EMw is solely reflecting changes in QT prolongation” (Stams et al., 2014): Dofetilide, well known to be a selective I_Kr_ blocker, exerts little or no effect on the Ca^2+^ transient, but it prolongs the APD_90_, thus causing, in turn, EMw shortening (Figure S4). However, our results suggest that the EMw is a better biomarker than APD_90_ for drugs with a multichannel effect, as it also reflects changes in the Ca^2+^ transient. It captures the balance between I_Kr_ and I_CaL_ blocks, which could result in a reduced likelihood of RA, while still causing APD_90_ prolongation (Figure S6). In addition, it is known that in the CAVB model, there is compensated hypertrophy and increased contractility, and numerous modifications in ion channels and Ca^2+^ handling have been reported, confirming a reduced repolarisation reserve (Bourgonje et al., 2013; Oros, Beekman, & Vos, 2008; Sipido et al., 2000; Volders et al., 1998). This could mask the effects of test article inhibition on inward currents (I_CaL_ and I_Na_) which could lead to a greater effect of pure hERG blockers and to a reduced ability to assess multichannel block.

The results of this study are in overall agreement with experimental EMw measurements obtained in the in vivo guinea pig model for 26 compounds, and recently published by Morissette et al. (2016). In that paper, the authors classified compounds in four classes: pro‐arrhythmic agents, rare cases of arrhythmias, negative controls, and positive inotropes. Compounds in the first two categories tend to induce EMw shortening, while compounds classified as negative controls show EMw increase or very little change following drug application. As mentioned above, this class also includes nicardipine and ivabradine. Results in the in vivo guinea pig are in qualitative agreement with our simulations, with the exception of the three positive inotropes: dobutamine, milrinone and levosimendan. These compounds are correctly classified as safe using our in silico trials, as they show a slight increase of the EMw following drug application. On the contrary, in the in vivo guinea pig model, they cause a substantial EMw shortening (>−10%), even though they have no effect on QTc interval and they are not classified as pro‐arrhythmic agents. This is because the shortening of the EMw induced by these compounds is not driven by QT prolongation (Morissette et al., 2016), but rather by other mechanisms. Milrinone and dobutamine act by increasing cAMP which ultimately increases intracellular Ca^2+^_,_ and in turn contractility, while levosimendan acts by increasing the affinity of troponin C for Ca^2+^, thus suggesting that the EMw can also shorten following an increase in contractility not necessarily related to an increase in intracellular Ca^2+^ concentration. We did not include those mechanisms in our in silico models, and therefore, they did not yielded the EMw shortening observed experimentally. The EMw considered in this study (CTD_90_–APD_90_) is a single cell surrogate measure for the in vivo EMw, defined as the difference between the end of the left ventricular pressure wave and the end of repolarisation (QT interval). In our study, we evaluate the effects of ion channel block on the EMw, as IC_50_ values are routinely evaluated during the drug development process. Therefore, the EMw only captures drug‐induced changes on ionic currents, in turn affecting Ca^2+^ transient. Extensions to this approach, such as the consideration of drugs affecting the contractile machinery directly, and not via electrophysiology, could be simulated by integrating cardiac contraction models available in literature (Land et al., 2017; Negroni & Lascano, 2008) and conducting electromechanical simulations, typically requiring supercomputing power, which would allow computation of biomarkers such as QT and left ventricular pressure. This would also improve EMw predictions for the positive inotropic compounds (i.e., dobutamine, milrinone, and levosimendan), bringing our simulation results closer to the experimental observations (Morissette et al., 2016). In addition, there are other Ca^2+^ biomarkers described, which could provide similar or complementary information to the EMw, for example, the rate of Ca^2+^ transient decay, shown to be slower in failing human ventricular myocytes (Piacentino et al., 2003). Finally, we considered drug effects on Na^+^, Ca^2+^, and K^+^ ion channels as inputs, and the EMw proved to be an effective biomarker to predict arrhythmic risk based on those. However, some drugs could affect other mechanisms, for example, SERCA pump, Na^+^–K^+^ pump, or Na^+^–Ca^2+^ exchanger, and should this information become available, it could be easily incorporated into our simulations.

To conclude, this study demonstrates that in silico drug trials using the EMw constitute a powerful methodology to predict clinical risk of arrhythmias based on ion channel information. Such information is frequently available at the early stages of lead compound identification, and the integration of computer models in the existing pipelines for drug safety assessment could lead to a major replacement of animal experiments in the preclinical stages of drug development.

## AUTHOR CONTRIBUTIONS

All the authors conceived and designed the study; E.P. designed the population of models for in silico drug assays, analysed the data, prepared the figures, and drafted the manuscript; E.P. and C.T. performed the simulations; P.M. and F.S. collected and provided the data for the reference compounds; E.P., A.B.O., and B.R. interpreted the results; all the authors edited and revised the manuscript and approved the final version.

## CONFLICT OF INTEREST

E.P., C.T., A.B.O., and B.R. declare no conflicts of interest. P.M. and F.S. are employees of Merck & Co., Inc.

## DECLARATION OF TRANSPARENCY AND SCIENTIFIC RIGOUR

This Declaration acknowledges that this paper adheres to the principles for transparent reporting and scientific rigour of preclinical research as stated in the *BJP* guidelines for Design & Analysis, and as recommended by funding agencies, publishers and other organisations engaged with supporting research.

## Supporting information


**Figure S1.** Drug‐induced % EMw changes (∆EMw) in the in silico population of 107 human models compared with control, for the 40 reference compounds tested at 1x and 3x EFTPC_max_ (Panels A and B, respectively). Figure description as in Figure 3.
**Figure S2.** Drug‐induced % EMw changes (∆EMw) in the in silico population of 107 human models compared with control, for the 40 reference compounds tested at 30x and 100x EFTPC_max_ (Panels A and B, respectively). Figure description as in Figure 3.
**Figure S3.** Comparison of the TdP score based on RA only in linear (top) and logarithmic (bottom) scale. Separation between risky and safe drugs is self‐evident only when showing the results in a logarithmic scale. For drugs with TdP score equal to 0, log_10_(0) was approximated with the machine precision (10^–16^).
**Figure S4.** Comparison of the APD_90_, CTD_90_ and EMw changes induced by different combinations of I_Kr_, I_CaL_ and I_NaL_ blocks, all causing the same APD_90_ prolongation (+44%) compared to control conditions, in the original ORd model: i) control (black); ii) 50% I_Kr_ block (pink); iii) 58% I_Kr_ block and 50% I_CaL_ block (blue); iv) 55% I_Kr_ block and 50% I_NaL_ block (green). Both ii) and iv) modify the APD_90_ with very little effect on CTD_90_ (Panel A), thus inducing a shortening of the EMw (Panel B). On the contrary, iii) has the same effect on APD_90_ but, due to the reduction in Ca^2^^+^ transient peak, which in turn causes a CTD_90_ prolongation, the EMw values remain almost identical to control.
**Figure S5.** This figure contains a summary of the sensitivity analysis results presented in Figure 7, but with I_CaL_ and I_NaL_ blocks swapped. Here, each column represents a different degree of I_CaL_ block, while each plot contains the 25 combinations of I_Kr_ and I_NaL_ blocks.
**Figure S6.** I_CaL_ block inhibits drug‐induced RA. The baseline ORd model (black traces) displays RA in presence of 85% I_Kr_ block (pink traces). A concomitant 10% I_CaL_ block (green traces) is enough to suppress RA, although not reversing APD_90_ prolongation.
**Figure S7.** Concentration‐dependent relation between models displaying EMw shortening and RA occurrence, for 4 tested compounds. Each dot represents one control model. Models displaying EMw shortening beyond threshold or RA are coloured in blue and pink, respectively. All the models displaying RA at a set testing concentration, also display EMw shortening at lower testing doses, thus confirming the EMw as an effective biomarker at lower testing concentrations, compared to RA.

Table S1.
IC_50_ and Hill coefficient (h) values used as inputs for the 40 in silico drug trials. For each compound, the EFTPC_max_ and TdP risk category are also included. Data come from different sources, and all the references are listed in the table.
