# Supplementary material for: Drug‐induced shortening of the electromechanical window is an effective biomarker for in silico prediction of clinical risk of arrhythmias
Source: Br J Pharmacol. 2019 Sep 4;176(19):3819–33. doi: 10.1111/bph.14786 (PMC6780030; doi:10.1111/bph.14786)
Supplement: Supplementary file 1 — Figure S1. Drug‐induced % EMw changes (∆EMw) in the in silico population of 107 human models compared with control, for the 40 reference compounds tested at 1x and 3x EFTPCmax (Panels A and B, respectively). Figure description as in Figure 3. Figure S2. Drug‐induced % EMw changes (∆EMw) in the in silico population of 107 human models compared with control, for the 40 reference compounds tested at 30x and 100x EFTPCmax (Panels A and B, respectively). Figure description as in Figure 3. Figure S3. Comparison of the TdP score based on RA only in linear (top) and logarithmic (bottom) scale. Separation between risky and safe drugs is self‐evident only when showing the results in a logarithmic scale. For drugs with TdP score equal to 0, log10(0) was approximated with the machine precision (10–16). Figure S4. Comparison of the APD90, CTD90 and EMw changes induced by different combinations of IKr, ICaL and INaL blocks, all causing the same APD90 prolongation (+44%) compared to control conditions, in the original ORd model: i) control (black); ii) 50% IKr block (pink); iii) 58% IKr block and 50% ICaL block (blue); iv) 55% IKr block and 50% INaL block (green). Both ii) and iv) modify the APD90 with very little effect on CTD90 (Panel A), thus inducing a shortening of the EMw (Panel B). On the contrary, iii) has the same effect on APD90 but, due to the reduction in Ca2 + transient peak, which in turn causes a CTD90 prolongation, the EMw values remain almost identical to control. Figure S5. This figure contains a summary of the sensitivity analysis results presented in Figure 7, but with ICaL and INaL blocks swapped. Here, each column represents a different degree of ICaL block, while each plot contains the 25 combinations of IKr and INaL blocks. Figure S6. ICaL block inhibits drug‐induced RA. The baseline ORd model (black traces) displays RA in presence of 85% IKr block (pink traces). A concomitant 10% ICaL block (green traces) is enough to suppress RA, although not reversing AP [file BPH-176-3819-s001.pdf]

Drug-induced Shortening of the Electromechanical Window is an Effective Biomarker for *in Silico* Prediction of Clinical Risk of Arrhythmias

SUPPORTING INFORMATION

Ion Channel Inhibition Measurement

Measurement of I_{Kr}, I_{Ks} and I_{Na} channel activities: PatchXpress automated patch clamp

Experimental procedures for measurement of I_{Kr} and I_{Ks} with PatchXpress were previously described in (Trepakova et al., 2007; Zeng et al., 2008). Methods for the determination of activity on the cardiac Na^+ channel were previously described in (Penniman et al., 2010).

Ca^{2+} influx fluorescence assay

L-type Ca^{2+} channels, hCav1.2, composed of 3 Ca^{2+} channel subunits (α_{1C}, $\alpha_2\delta$, β_{2a}) (Balasubramanian et al., 2009) were stably expressed in HEK-293 cells, along with an inwardly-rectifying potassium channel, $hKir_{2.3}$, to set a more negative resting membrane potential (-65 mV at an external K^+ concentration $[K^+]_e$ = 5.8 mM) and promote depolarization of the resting membrane potential by raising $[K^+]_e$ (Xia et al., 2004), and thereby increasing the K^+ equilibrium potential. HEK-293 cells were grown in culture media containing DMEM 'Glutamax' (Invitrogen Corp.) supplemented with 10% Fetal Bovine Serum, 100 U/ml penicillin, and 100 µg/ml streptomycin, as well as selection antibiotics Geneticin G418 (100–800 µg/ml) for α1C, Zeocin (40 µg/ml) for Kir2.3, and Hygromycin B (100–250 µg/ml) for β2a. HEK-Cav1.2 cells were incubated at 37 °C in filtered ambient air supplemented with 5% CO_2. Cells were grown to approximately 70–80% confluency and passaged twice a week. All tissue culture reagents were obtained from Invitrogen Corp.

Prior to conducting assays, cells were seeded in poly-D-lysine coated 96-well plates (BioCoat) at 60 K cells per well and incubated for 24–48 h at 37 °C. On the day of assay conduct, cells were rinsed with Wash Buffer (containing in mM: 5.8 KCl, 146.2 NaCl, 0.005 $CaCl_2$, 1.7 $MgCl_2$, and 10 HEPES) and then incubated in Wash Buffer supplemented with 4 µM FLUO-4 AM, 0.02% Pluronic acid (both from Molecular Probes), and 10 mM D-Glucose for 30 min at room temperature in the dark. After this Ca^{2+}-sensitive dye loading step, cells were washed with and then kept in 0.05 mL Incubation Buffer (containing in mM: 25 KCl, 127 NaCl, 0.005 $CaCl_2$, 1.7 $MgCl_2$, and 10 HEPES) with test agent or positive or negative control drugs at various test concentrations for 30 min at room temperature in the dark. All buffers were adjusted to pH 7.2 with NaOH or HCL prior to use. The plate was subsequently placed in the reading chamber of a fluorimeter (FlexStation III, Molecular Devices) and the emission of each well at a wavelength of 535 nM was measured in response to excitation at a wavelength 480 nM at sampling intervals of 1.6 s.

Following a baseline reading for 10–20 s, Ca^{2+}-Trigger Buffer (containing in mM: 119 NaCl; 25 KCl; 4 mM $CaCl_2$; 1.7 $MgCl_2$ and 10 HEPES) was added at a volume equal to that of the Incubation Buffer already present

in the well, after which the emission intensity at 535 nM was measured for another 20–30 s. The Ca^{2+} influx signal was quantified as the difference between the baseline and the peak of the emission signal in response to the addition of Ca^{2+}-Trigger Buffer, which was typically reached within 20 s thereafter. The Ca^{2+} signal from each well containing test agent was normalized to a signal window defined as the difference between the positive (100% inhibition) and negative (0% inhibition) control signals.

Additional Drug Information

In addition to the 4 ion channels considered for all compounds, IC_{50}/h for Late Na^+ current (I_{NaL}) were included to simulate Mexiletine and Ranolazine action. One drug with possible TdP risk (Risperidone) and two drugs with known TdP risk (Amiodarone and Pentamidine), were correctly classified as risky only after considering information from literature, in addition to the IC_{50}/h for the ion channels. Risperidone and Amiodarone were already discussed in detail in (Passini et al., 2017). One of Risperidone metabolites (Paliperidone) strongly affects hERG channel, and patients taking Risperidone orally exhibit plasma level of Paliperidone up to 10 times higher than their phase level of Risperidone (Alamo and López-muñoz, 2013). Therefore, we combined the effect on ion channels of both Risperidone and Paliperidone in the values reported in Table S1. As for Amiodarone, its pro-arrhythmic side effects are often underestimated compared to other anti-arrhythmic drugs (Jurado Román et al., 2012). It has almost no effect on cardiac electrophysiology at the $EFTPC_{max}$ = 0.0008 µM reported in literature (Kramer et al., 2013): 0.06%, 0.05%, 0.09%, and 0.00% block for I_{Na}, I_{CaL}, I_{Kr} and I_{Ks}, respectively, based on the the IC_{50} and h used in this study. However, the $EFTPC_{max}$ reported by (Kramer et al., 2013) was computed based on 99.98% protein binding for Amiodarone, while values reported in literature range from 96% (Lalloz et al., 1984; Latini et al., 1984) to 99.98% (Veronese et al., 1988). When considering a lower protein binding, the $EFTPC_{max}$ can vary up to more than 100-fold, thus completely changing the safety profile of the drug from safe to risky. In this study, we chose to consider the worst case scenario: protein binding 96%, leading to $EFTPC_{max}$ = 0.155 µM, as shown in Table S1. The second known risk drug for which we had to include additional information is Pentamidine. Experimentally, Pentamidine was found to have no effect on three out of the four ion channels tested in this study (I_{CaL}, I_{Kr}, I_{Ks}), and a very small effect on I_{Na} ($EFTPC_{max}$ = 0.116 µM, IC_{50} = 53 µM), as shown in Table S1. However, Pentamidine is known to have a strong indirect effect on I_{Kr}, by inhibiting hERG channel trafficking and maturation (Kuryshev et al., 2005). Therefore, we took this effect into account in our simulations by using the I_{Kr} IC_{50} of 5.1 µM proposed by (Kuryshev et al., 2005), determined by analysing Pentamidine-induced changes in hERG tail current amplitudes.

False Negatives/Positives

Compounds misclassified as false negatives (Nicardipine, Ivabradine and Amitritpyline) all belong to potential/conditional risk categories, which are usually associated with overdoses or interactions with other drugs, and often controversial. As an example, two of our false negatives (Nicardipine and Ivabradine) are actually considered as negative control in (Morissette et al., 2016). Indeed, there is no TdP risk warning on

the FDA label for these drugs, nor strong evidence in the literature that they would cause TdP if taken alone and as recommended. Their main effect is via changes in heart rate (increased by Nicardipine, decreased by Ivabradine), leading to a corrected QT (QTc) prolongation for Nicardipine, and a QT (but not QTcs) prolongation for Ivabradine. Nicardipine was also classified as no TdP risk in (Champéroux et al., 2005) and no changes in QT were reported in patients exposed to the drug (Iribarren et al., 2013) nor in marmoset (Horii et al., 2002), except for one study with 293 patients (Sasaoka et al., 2016) of which 18 showed adverse events which might be broadly related to drug-induced long QT syndrome. As for Ivabradine, it is generally considered to have a good cardiac safety profile (Camm and Lau, 2003; Savelieva and Camm, 2006), and its conditional TdP risk seem to be indirect, i.e. depending on the decrease of heart rate or due to its combination with other QT prolonging drugs, even if it has also been shown to have a direct effect on the hERG channel (Hancox et al., 2015).

The third and last false negative, Amitriptyline, affects both I_{Kr} and I_{CaL} currents: therefore, the resulting safety profile is given by the balance between these two blocks, the first leading to QT prolongation and potentially TdP, and the latter contributing to QT shortening and suppression of RA. The magnitude of I_{Kr} block has been shown to be both voltage and use dependent, as well as depending on the external K^+ concentration (Jo et al., 2000), and there is evidence of Amytriptyline also affecting hERG trafficking after prolonged drug exposure (Dennis et al., 2011). IC_{50} reported in literature for I_{CaL} are contradictory, ranging from 0.57 μM in this study, to 1.29 μM in (Crumb et al., 2016) and up to 23.2 μM measured in rat cardiomyocytes (Zahradnı et al., 2008), the latter also used in two previously published *in silico* studies (Mirams et al., 2011; Lancaster and Sobie, 2016) to predict drug TdP risk. This suggests that our input data might minimize Amitriptyline risk, by underestimating I_{Kr} block and overestimating I_{CaL} block, thus making it a false negative.

The only compound misclassified as false positive in this study is Mexiletine, a multi-channel blocker, affecting mainly fast and late I_{Na}, as well as I_{Kr}. We can speculate that the main reason for the misclassification relies in an overestimation of I_{Kr} block when using a simple pore drug block model. Indeed, there is evidence of Mexiletine binding preferably to the open state of the hERG channel (Gualdani et al., 2015), and therefore a dynamic hERG channel drug block model such as the one recently proposed by the FDA (Dutta et al., 2017; Li et al., 2017) could improve predictions. The main reasons for using the original ORd human ventricular model in this study, rather than its updated version, are the lack of experimental data on dynamic hERG block for our reference compounds, and the possibility of comparison with previous results. Additional reasons for the use of the ORd model are included in (Passini et al., 2017). In addition, Mexiletine has many known metabolites, most of them with no pharmacological effect (Labbé and Turgeon, 1999), or in some cases with the same I_{Na} block properties, but less effect on I_{Kr} (Catalano et al., 2012; Gualdani et al., 2015). This information is not currently taken into account in our model, and could improve risk predictions.

REFERENCES

Alamo, C., and López-muñoz, F. (2013). The Pharmacological Role and Clinical Applications of Antipsychotics ' Active Metabolites : Paliperidone versus Risperidone. Clin. Exp. Pharmacol. *3*: 1–12.

Balasubramanian, B., Imredy, J.P., Kim, D., Penniman, J., Lagrutta, A., and Salata, J.J. (2009). Optimization of Cav1.2 screening with an automated planar patch clamp platform. J. Pharmacol. Toxicol. Methods *59*: 62–72.

Camm, A.J., and Lau, C.-P. (2003). Electrophysiological effects of a single intravenous administration of ivabradine (S 16257) in adult patients with normal baseline electrophysiology. Drugs R. D. *4*: 83–9.

Catalano, A., Desaphy, J.-F., Lentini, G., Carocci, A., Mola, A. Di, Bruno, C., et al. (2012). Synthesis and Toxicopharmacological Evaluation of *m* -Hydroxymexiletine, the First Metabolite of Mexiletine More Potent Than the Parent Compound on Voltage-Gated Sodium Channels. J. Med. Chem. *55*: 1418–1422.

Champéroux, P., Viaud, K., Amrani, A.I. El, Fowler, J.S.L., Martel, E., Guennec, J.-Y. Le, et al. (2005). Prediction of the risk of Torsade de Pointes using the model of isolated canine Purkinje fibres. Br. J. Pharmacol. *144*: 376–385.

Crumb, W.J., Vicente, J., Johannesen, L., and Strauss, D.G. (2016). An evaluation of 30 clinical drugs against the comprehensive in vitro proarrhythmia assay (CiPA) proposed ion channel panel. J. Pharmacol. Toxicol. Methods *81*: 251–262.

Dennis, A.T., Nassal, D., Deschenes, I., Thomas, D., and Ficker, E. (2011). Antidepressant-induced ubiquitination and degradation of the cardiac potassium channel hERG. J. Biol. Chem. *286*: 34413–25.

Dutta, S., Chang, K.C., Beattie, K.A., Sheng, J., Tran, P.N., Wu, W.W., et al. (2017). Optimization of an In silico Cardiac Cell Model for Proarrhythmia Risk Assessment. Front. Physiol. *8*: 616.

Gualdani, R., Tadini-Buoninsegni, F., Roselli, M., Defrenza, I., Contino, M., Colabufo, N.A., et al. (2015). Inhibition of hERG potassium channel by the antiarrhythmic agent mexiletine and its metabolite m-hydroxymexiletine. Pharmacol. Res. Perspect. *3*: e00160.

Hancox, J.C., Melgari, D., Dempsey, C.E., Brack, K.E., Mitcheson, J., and Ng, G.A. (2015). hERG potassium channel inhibition by ivabradine may contribute to QT prolongation and risk of torsades de pointes. Ther. Adv. Drug Saf. *6*: 177–9.

Horii, I., Kito, G., Hamada, T., Jikuzono, T., Kobayashi, K., and Hashimoto, K. (2002). Development of telemetry system in the common marmoset - Cardiovascular effects of astemizole and nicardipine. J. Toxicol. Sci. *27*: 123–130.

Iribarren, C., Round, A.D., Peng, J.A., Lu, M., Zaroff, J.G., Holve, T.J., et al. (2013). Validation of a population-based method to assess drug-induced alterations in the QT interval: A self-controlled crossover study. Pharmacoepidemiol. Drug Saf. *22*: 1222–1232.

Jo, S.H., Youm, J.B., Lee, C.O., Earm, Y.E., and Ho, W.K. (2000). Blockade of the HERG human cardiac K(+) channel by the antidepressant drug amitriptyline. Br. J. Pharmacol. *129*: 1474–80.

Jurado Román, A., Rubio Alonso, B., Martín Asenjo, R., Salguero Bodes, R., López Gil, M., and Arribas Ynsaurriaga, F. (2012). Proarrhythmic Potential of Amiodarone: An Underestimated Risk? Rev. Española Cardiol. (English Ed. *65*: 292–294.

Kramer, J., Obejero-Paz, C.A., Myatt, G., Kuryshev, Y.A., Bruening-Wright, A., Verducci, J.S., et al. (2013). MICE Models: Superior to the HERG Model in Predicting Torsade de Pointes. Sci. Rep. *3*: 2100.

Kuryshev, Y.A., Ficker, E., Wang, L., Hawryluk, P., Dennis, A.T., Wible, B.A., et al. (2005). (27) Pentamidine-Induced Long QT Syndrome and Block of hERG Trafficking. J. Pharmacol. Exp. Ther. *312*: 316–323.

Labbé, L., and Turgeon, J. (1999). Clinical Pharmacokinetics of Mexiletine. Clin. Pharmacokinet. *37*: 361–384.

Lancaster, M.C., and Sobie, E.A. (2016). Improved Prediction of Drug-Induced Torsades de Pointes Through Simulations of Dynamics and Machine Learning Algorithms. Clin. Pharmacol. Ther. *100*: 371–379.

Li, Z., Dutta, S., Sheng, J., Tran, P.N., Wu, W., Chang, K., et al. (2017). Improving the In Silico Assessment of Proarrhythmia Risk by Combining hERG (Human Ether-à-go-go-Related Gene) Channel-Drug Binding Kinetics and Multichannel Pharmacology. Circ. Arrhythmia Electrophysiol. *10*:.

Mirams, G.R., Cui, Y., Sher, A., Fink, M., Cooper, J., Heath, B.M., et al. (2011). Simulation of multiple ion channel block provides improved prediction of compounds' clinical torsadogenic risk. Cardiovasc. Res. *91*: 53–61.

Morissette, P., Regan, C., Fitzgerald, K., Gerenser, P., Travis, J., Wang, S., et al. (2016). Shortening of the electromechanical window in the ketamine/xylazine-anesthetized guinea pig model to assess pro-arrhythmic risk in early drug development. J. Pharmacol. Toxicol. Methods *81*: 171–182.

Passini, E., Britton, O.J., Lu, H.R., Rohrbacher, J., Hermans, A.N., Gallacher, D.J., et al. (2017). Human In Silico Drug Trials Demonstrate Higher Accuracy than Animal Models in Predicting Clinical Pro-Arrhythmic Cardiotoxicity. Front. Physiol. *8*: 1–15.

Penniman, J.R., Kim, D.C., Salata, J.J., and Imredy, J.P. (2010). Assessing use-dependent inhibition of the cardiac Na± current (INa) in the PatchXpress automated patch clamp. J. Pharmacol. Toxicol. Methods *62*: 107–118.

Savelieva, I., and Camm, A.J. (2006). Novel If current inhibitor ivabradine: safety considerations. Adv. Cardiol. *43*: 79–96.

Trepakova, E.S., Malik, M.G., Imredy, J.P., Penniman, J.R., Dech, S.J., and Salata, J.J. (2007). Application of PatchXpress planar patch clamp technology to the screening of new drug candidates for cardiac KCNQ1/KCNE1 (I Ks) activity. Assay Drug Dev. Technol. *5*: 617–27.

Xia, M., Imredy, J.P., Koblan, K.S., Bennett, P., and Connolly, T.M. (2004). State-dependent inhibition of L-type calcium channels: Cell-based assay in high-throughput format. Anal. Biochem. *327*: 74–81.

Zahradnı, I., Minarovic, I., Zahradnı, A., Zahradník, I., Minarovic, I., and Zahradníková, A. (2008). Inhibition of the Cardiac L-Type Calcium Channel Current by Antidepressant Drugs. Pharmacol. Exp. Ther. *324*: 977–984.

Zeng, H., Penniman, J.R., Kinose, F., Kim, D., Trepakova, E.S., Malik, M.G., et al. (2008). Improved Throughput of PatchXpress hERG Assay Using Intracellular Potassium Fluoride. Assay Drug Dev. Technol. *6*: 235–241.

SUPPORTING FIGURES

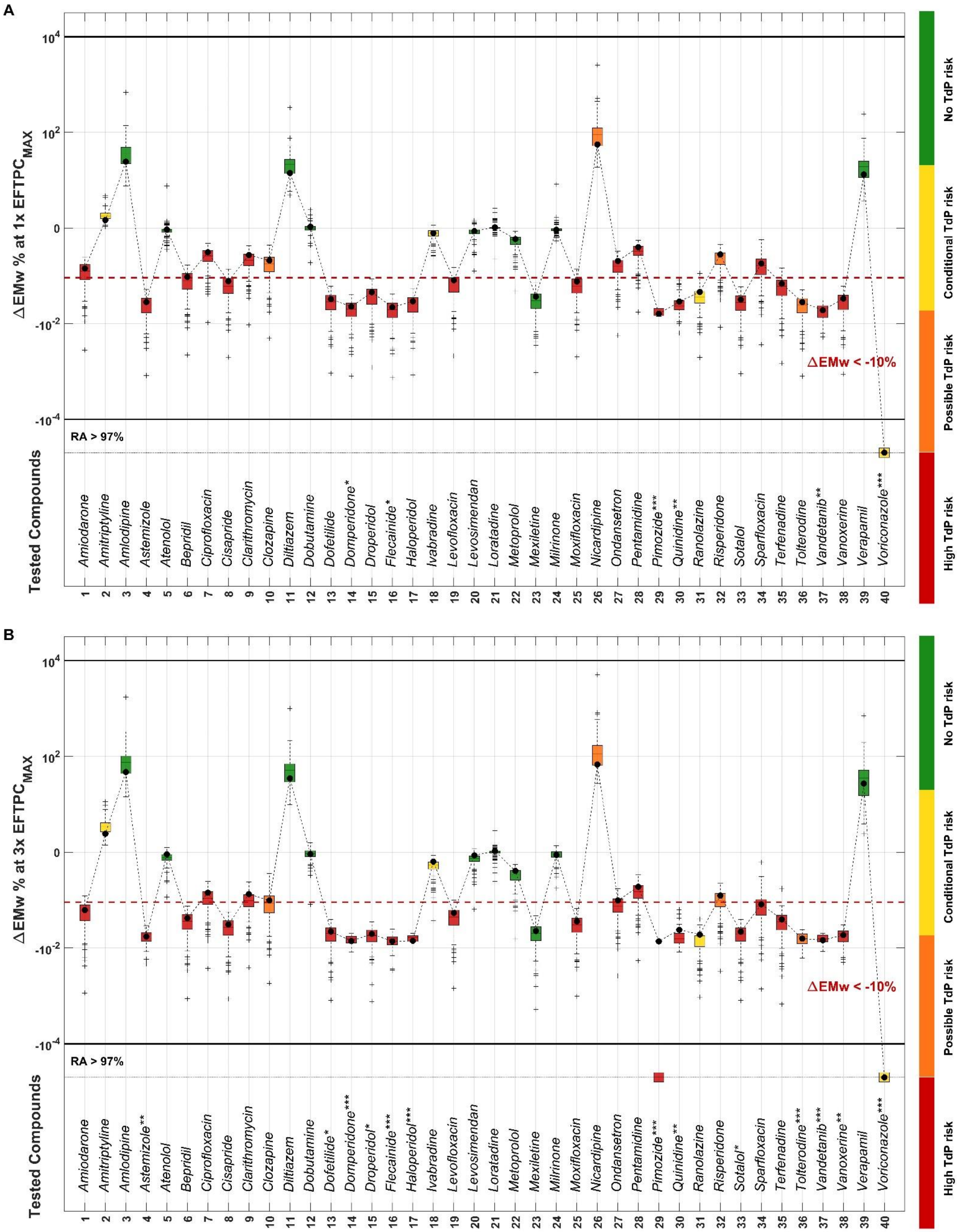

Figure 1S. Drug-induced % EMw changes (ΔEMw) in the in silico population of 107 human models compared to control, for the 40 reference compounds tested at 1x and 3x $EFTPC_{max}$ (Panels A and B, respectively). Figure description as in Figure 3.

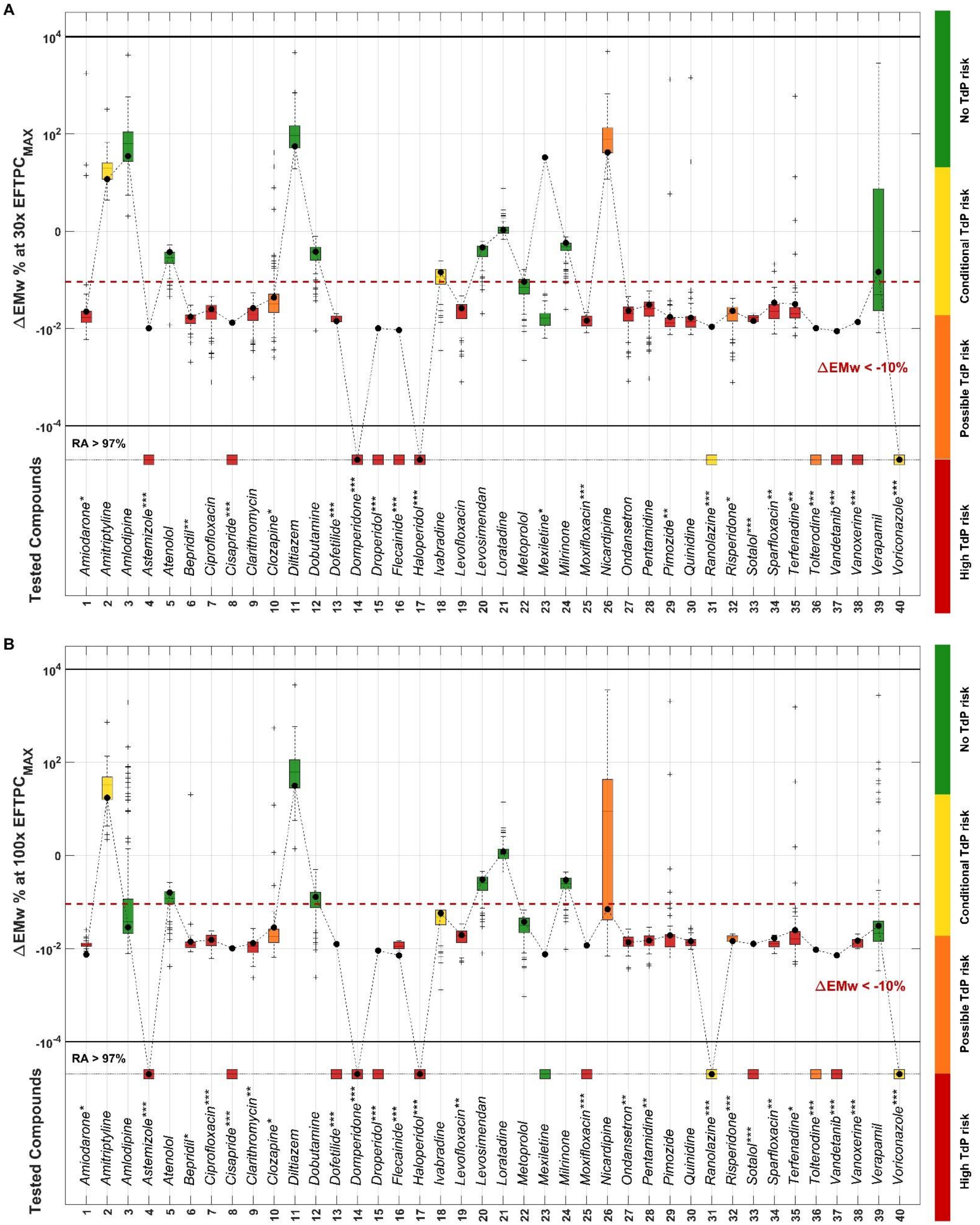

Figure 2S. Drug-induced % EMw changes (ΔEMw) in the in silico population of 107 human models compared to control, for the 40 reference compounds tested at 30x and 100x $EFTPC_{max}$ (Panels A and B, respectively). Figure description as in Figure 3.

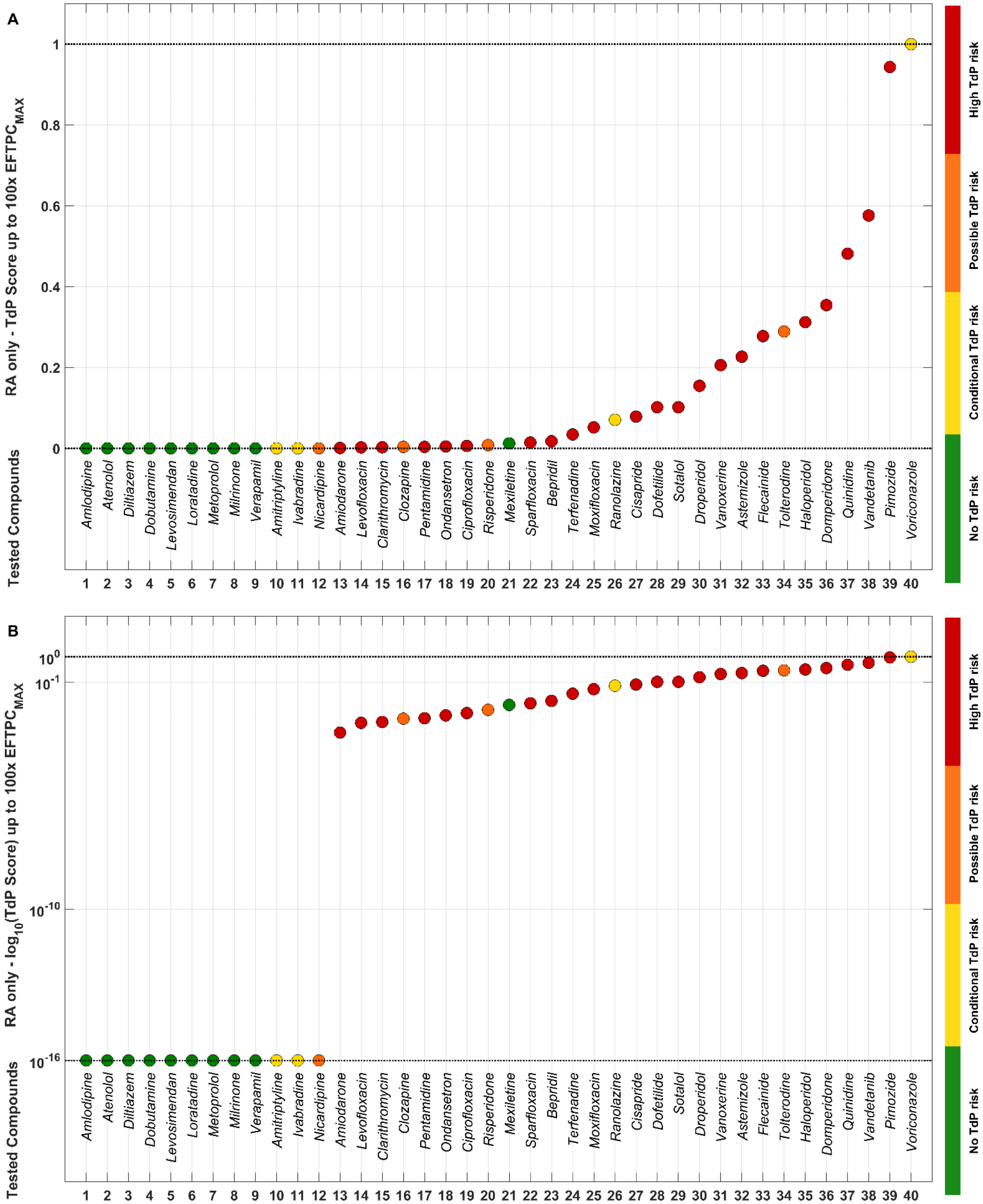

Figure 3S. Comparison of the TdP score based on RA only in linear (top) and logarithmic (bottom) scale. Separation between risky and safe drugs is self-evident only when showing the results in a logarithmic scale. For drugs with TdP score equal to 0, $\log_{10}(0)$ was approximated with the machine precision (10^{-16}).

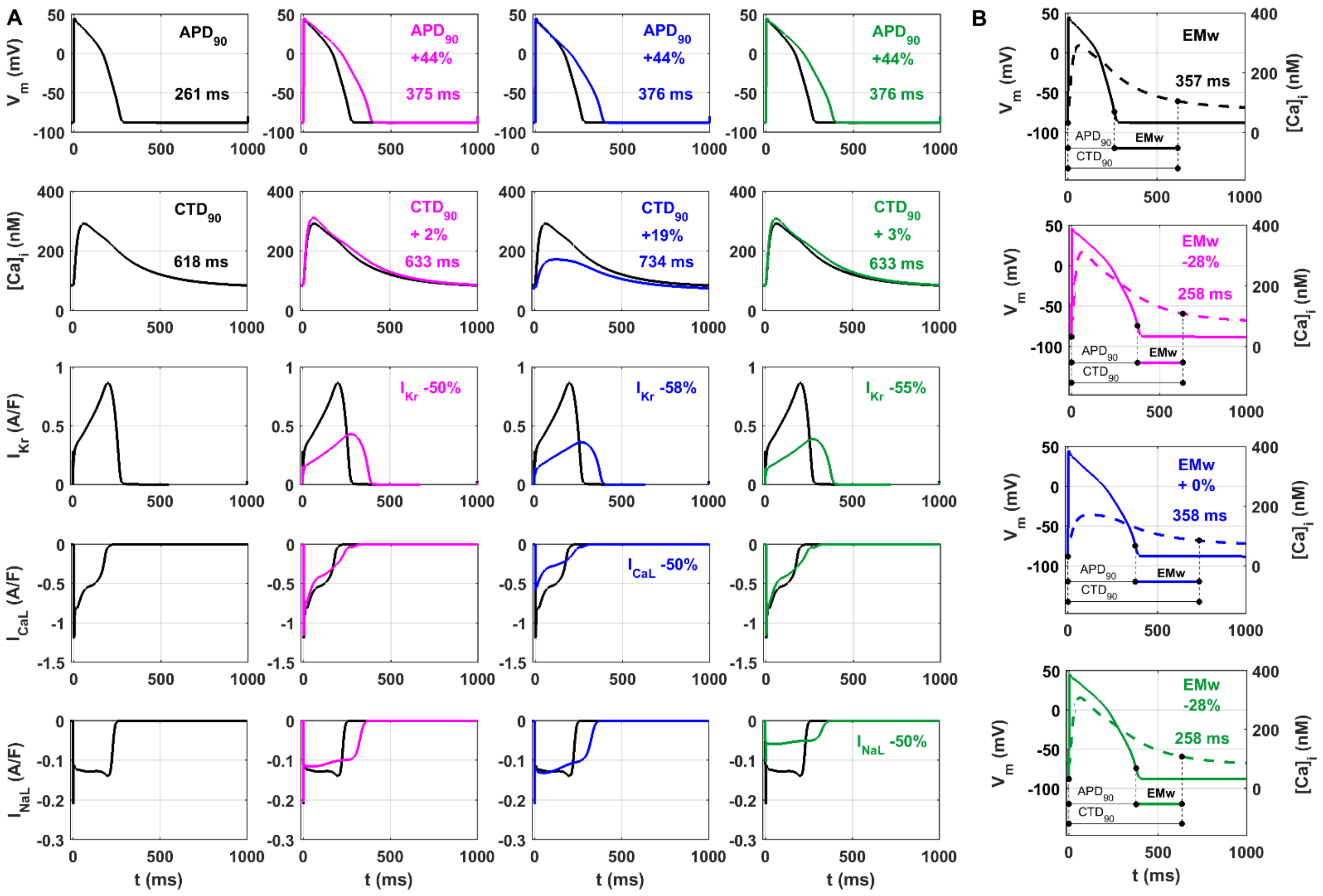

Figure 4S. Comparison of the APD_{90}, CTD_{90} and EMw changes induced by different combinations of I_{Kr}, I_{CaL} and I_{NaL} blocks, all causing the same APD_{90} prolongation (+44%) compared to control conditions, in the original ORd model: i) control (black); ii) 50% I_{Kr} block (pink); iii) 58% I_{Kr} block and 50% I_{CaL} block (blue); iv) 55% I_{Kr} block and 50% I_{NaL} block (green). Both ii) and iv) modify the APD_{90} with very little effect on CTD_{90} (Panel A), thus inducing a shortening of the EMw (Panel B). On the contrary, iii) has the same effect on APD_{90} but, due to the reduction in Ca^{2+} transient peak, which in turn causes a CTD_{90} prolongation, the EMw values remain almost identical to control.

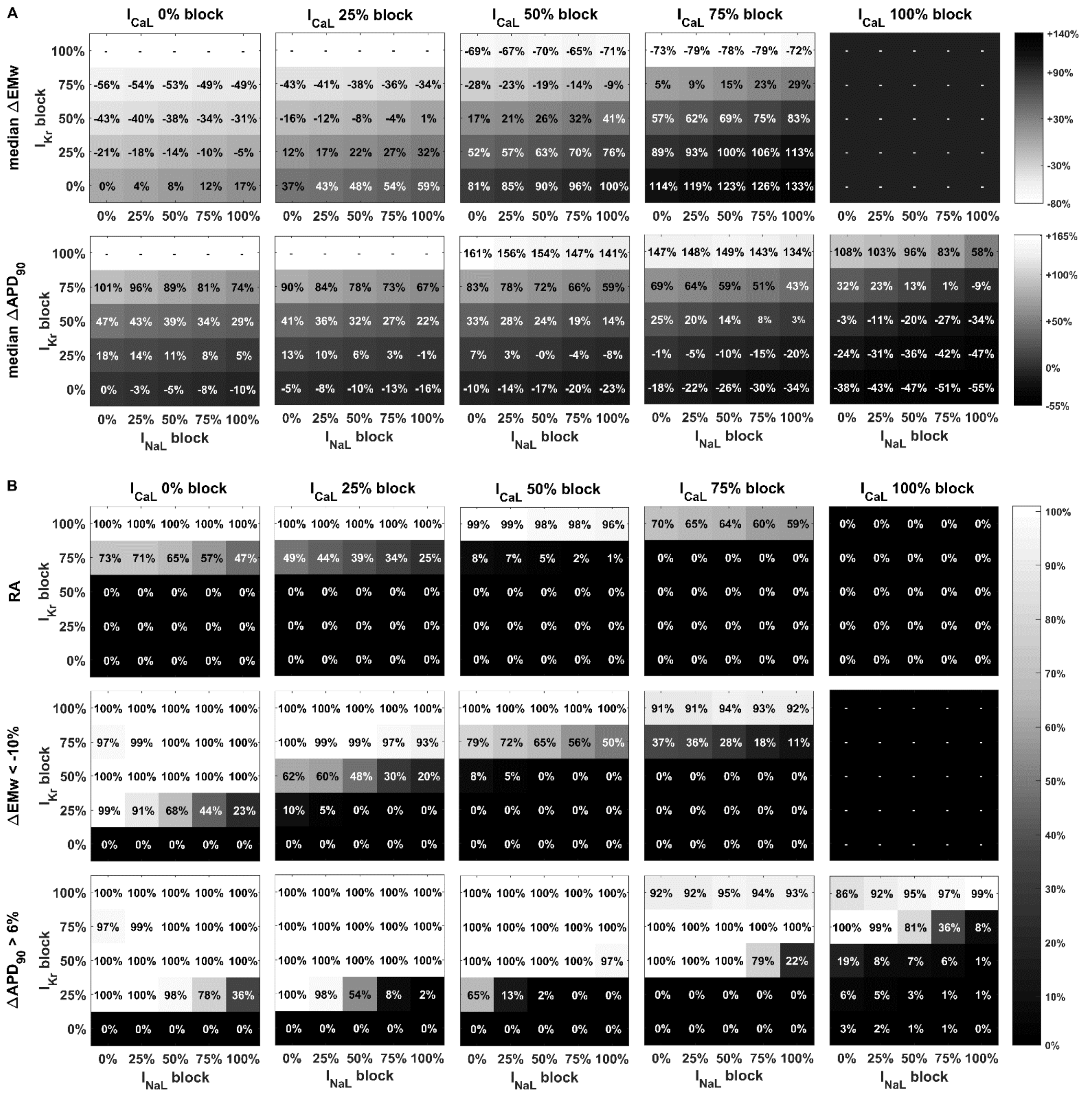

Figure 5S. This figure contains a summary of the sensitivity analysis results presented in Figure 7, but with I_{CaL} and I_{NaL} blocks swapped. Here, each column represents a different degree of I_{CaL} block, while each plot contains the 25 combinations of I_{Kr} and I_{NaL} blocks.

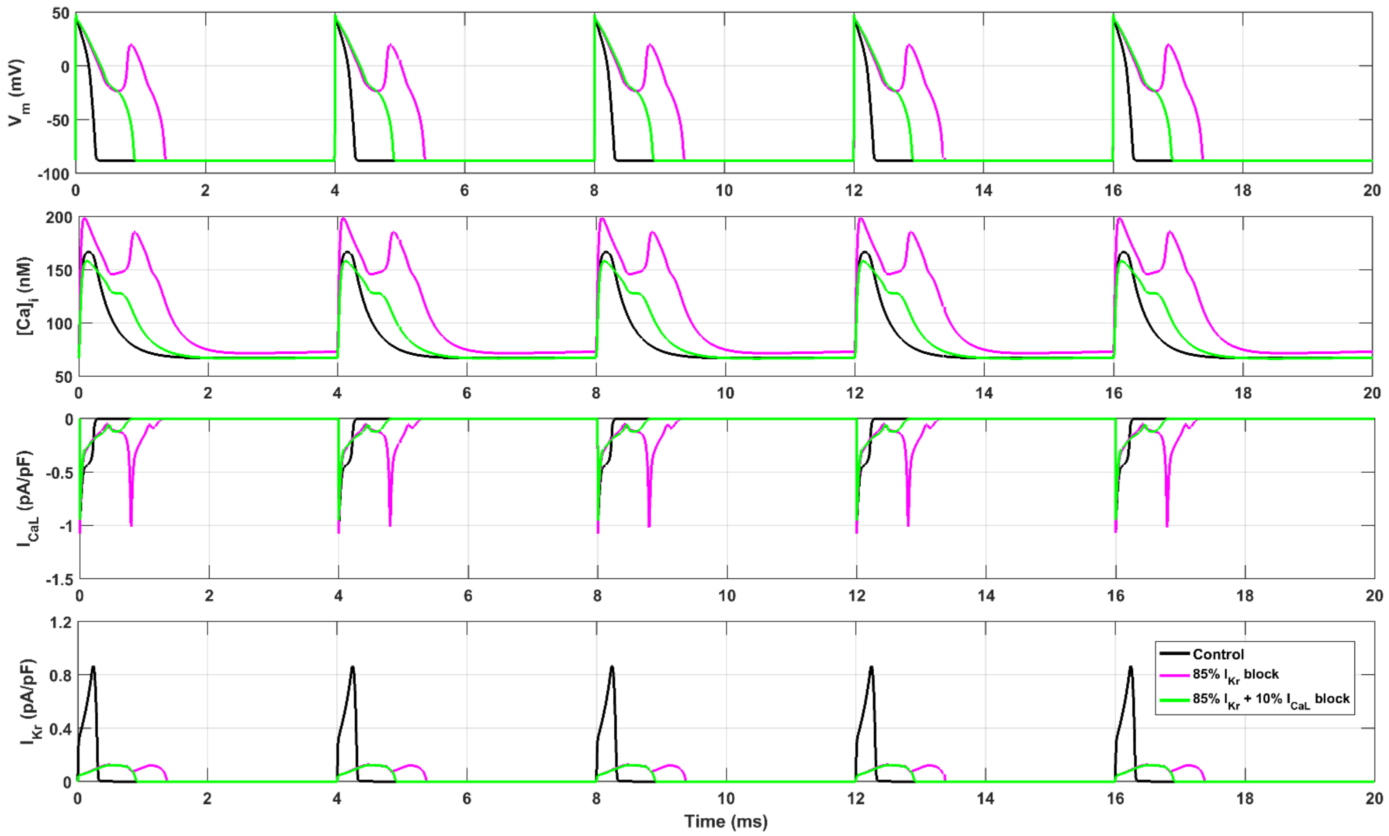

Figure 6S. I_{CaL} block inhibits drug-induced RA. The baseline ORd model (black traces) displays RA in presence of 85% I_{Kr} block (pink traces). A concomitant 10% I_{CaL} block (green traces) is enough to suppress RA, although not reversing APD_{90} prolongation.

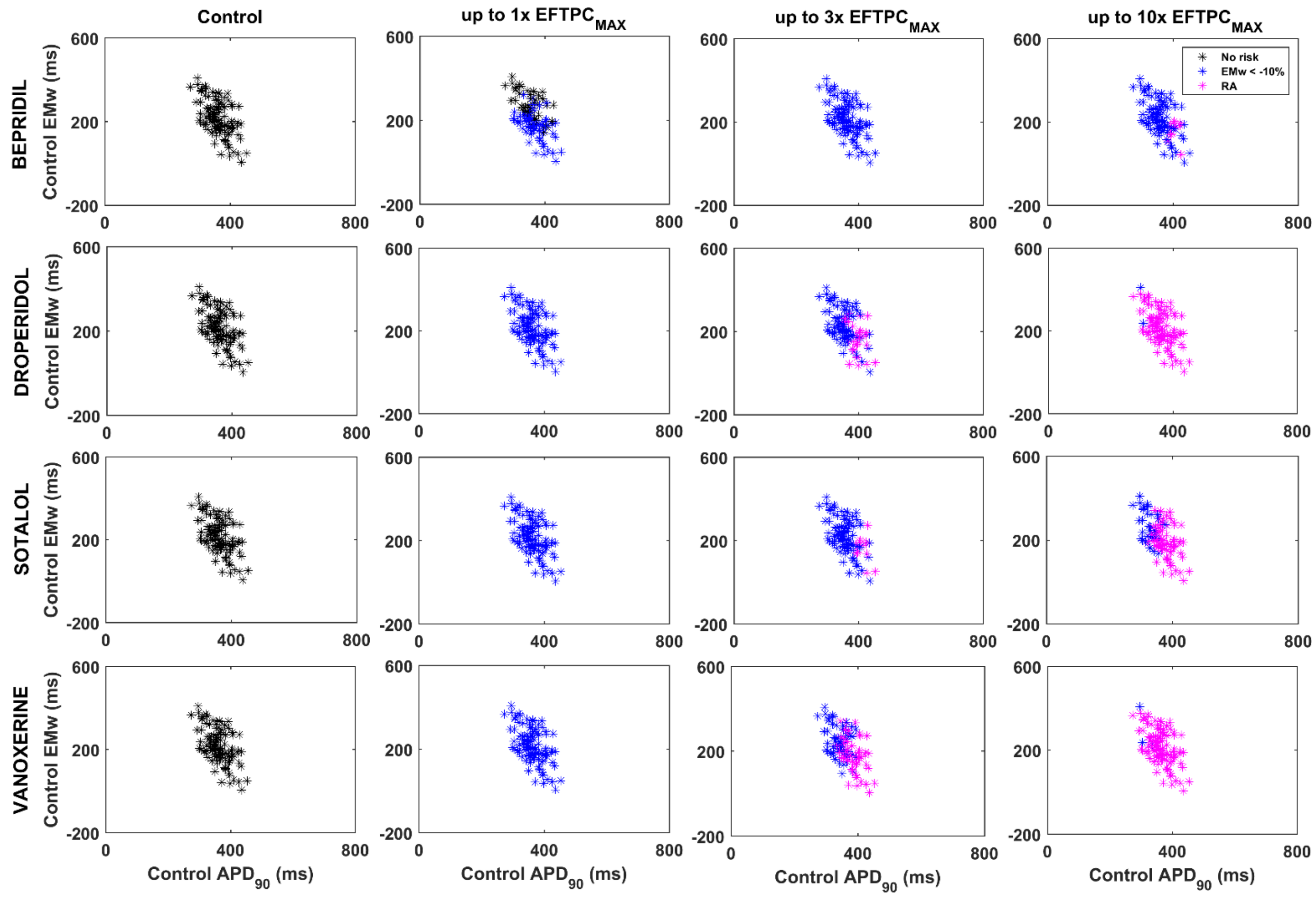

Figure 7S. Concentration-dependent relation between models displaying EMw shortening and RA occurrence, for 4 tested compounds. Each dot represents one control model. Models displaying EMw shortening beyond threshold or RA are coloured in blue and pink, respectively. All the models displaying RA at a set testing concentration, also display EMw shortening at lower testing doses, thus confirming the EMw as an effective biomarker at lower testing concentrations, compared to RA.
