# Supplementary material for: Drug‐induced shortening of the electromechanical window is an effective biomarker for in silico prediction of clinical risk of arrhythmias
Source: Br J Pharmacol. 2019 Sep 4;176(19):3819–33. doi: 10.1111/bph.14786 (PMC6780030; doi:10.1111/bph.14786)
Supplement: Supplementary file 2 — Table S1. IC50 and Hill coefficient (h) values used as inputs for the 40 in silico drug trials. For each compound, the EFTPCmax and TdP risk category are also included. Data come from different sources, and all the references are listed in the table. [file BPH-176-3819-s002.pdf]

Table S1. IC_{50} and Hill coefficient (h) values used as inputs for the 40 *in silico* drug trials. For each compound, the $EFTPC_{max}$ and TdP risk category are also included. Data come from different sources, and all the references are listed in the table.

Compound List		$IC_{50}(h)^{REF}$, with IC_{50} in μM					EFTPCmax (μM)	TdP risk category[†]	Drug also included in Passini et al. 2017[7]
		I_{Na}	I_{CaL}	I_{Kr}	I_{Ks}	I_{NaL}			
1	Amiodarone	1.312(1)[1]	1.545(1)[1]	0.86(1)[1]	17.6(1)[1]		0.155 [#]	1	X
2	Amitriptyline	0.674(1)[1]	0.5683(1)[1]	1.8(1)[1]	48.387(0.89)[1]		0.0086 [*]	3	
3	Amlodipine	5.942(1)[1]	0.315(1)[1]	2.6(1.33)[1]	5.9(1.65)[1]		0.105 [*]	0	
4	Astemizole	1.799(1)[1]	1.4(1)[1]	0.00035(0.9)[1]	10(2.7)[1]		0.0003 [5]	1	X
5	Atenolol			1500(0.92)[1]			1.156 [*]	0	
6	Bepridil	0.6267(1)[1]	0.6753(1)[1]	0.16(1)[1]	5.5(1.76)[1]		0.035 [5]	1	X
7	Ciprofloxacin			335(1)[1]			12.072 [*]	1	
8	Cisapride	42.857(1)[1]	3.25(1)[1]	0.01(1.1)[1]	68.182(2.55)[1]		0.0026 [5]	1	X
9	Clarithromycin	163.576(1)[1]		62.5(0.84)[1]			1.337 [*]	1	
10	Clozapine	10.22(1)[1]	3.108(1)[1]	0.88(0.94)[1]	19(1.52)[1]		0.071 [5]	2	X
11	Diltiazem	18.85(1)[1]	0.653(1)[1]	7.5(0.95)[1]			0.122 [5]	NC	X
12	Dobutamine	117.187(1)[1]	55.147(1)[1]	15(1.17)[1]	53.571(2.28)[1]		0.03819 [*]	0	
13	Dofetilide		100(1)[1]	0.0037(0.6)[1]			0.002 [3,5]	1	X
14	Domperidone	6.322(1)[1]	50(1)[1]	0.046(1.27)[1]			0.055 [*]	1	
15	Droperidol	1.819(1)[1]	4.889(1)[1]	0.08(1.2)[1]			0.04 [*]	1	X
16	Flecainide	6.995(1)[1]	27(1)[1]	0.61(0.93)[1]			0.752 [3,5]	1	X
17	Haloperidol	1.113(1)[1]	1.7(1)[1]	0.0028(1.7)[1]	22(1.4)[1]		0.0025 [*]	1	X
18	Ivabradine	78.947(1)[1]	34.091(1)[1]	3.6(0.99)[1]			0.014 [5]	3	
19	Levofloxacin			660(0.46)[1]			22.26 [*]	1	
20	Levosimendan	85.714(1)[1]	28.45(1)[1]	22(0.68)[1]			0.0028 [*]	0	
21	Loratadine	4.722(1)[1]	10.21(1)[1]	6.2(1.7)[1]	10.2(4.6)[1]		0.0015 [*]	0	X
22	Metoprolol			113(0.86)[1]	88.235(1.54)[1]		0.5 [*]	0	
23	Mexiletine	11.22(1)[1]	22.09(1)[1]	4.1(0.78)[1]		8.957(1.4)[3]	2.5 [3]	NC	X
24	Milrinone			1500(0.85)[1]			0.316 [*]	0	
25	Moxifloxacin			42(0.9)[1]			8 [*]	1	X
26	Nicardipine	1.02(1)[1]	0.0097(1)[1]	0.3(1.11)[1]			0.014 [*]	2	
27	Ondansetron	12.97(1)[1]		1.1(0.8)[1]			0.038 [*]	1	
28	Pentamidine	52.687(1)[1]		5.1(1)[##]			0.116 [*]	1	
29	Pimozide	0.5742(1)[1]	0.027(1)[1]	0.00084(1.15)[1]	2.7(1.2)[1]		0.004 [*]	1	X
30	Quinidine	51.493(1)[1]	6.4(1)[1]	0.95(1)[1]	270(1)[1]		3.237 [5]	1	X
31	Ranolazine	11.18(1)[1]		6(1.04)[1]		7.887(0.9)[3]	1.95 [3]	3	X
32	Risperidone[N]	55.46(1)[1]	15.25(1)[1]	0.046(1)[1]	136.364(2.12)[1]		0.002 [5]	2	X
33	Sotalol			26(0.58)[1]			14.69 [5]	1	X
34	Sparfloxacin		72(1)[1]	16.3(1)[1]	57.692(1)[1]		1.766 [5]	1	X
35	Terfenadine	2.044(1)[1]	0.2(1)[1]	0.031(0.88)[1]	4.2(2.15)[1]		0.009 [5]	1	X
36	Tolterodine	4.675(1)[1]	1.4(1)[1]	0.0071(1.24)[1]			0.0065 [*]	2	
37	Vandetanib	53.571(1)[1]	19.72(1)[1]	0.123(0.9)[1]	20(1)[1]		0.253 [*]	1	
38	Vanoxerine	1.7(0.746)[1]	0.363(1)[1]	0.011(1.06)[1]	12(2.64)[1]		0.008 [9]	1	
39	Verapamil	7.412(1)[1]	0.161(1)[1]	0.25(1.27)[1]	107.143(1)[1]		0.036 [*]	NC	X
40	Voriconazole			0.0118(1.5)[1]			7.563 [5]	3	X

† TdP Risk Category from CredibleMeds[7]

1 High TdP Risk: the drug prolongs the QT interval and is clearly associated with a known TdP risk, even when taken as recommended

2 Possible TdP Risk: the drug prolongs the QT interval, but there is a lack of evidence of TdP risk when taken as recommended

3 Conditional TdP Risk: the drug is associated with TdP but only under certain circumstances, e.g. excessive dose or interaction with other drugs

0 No TdP Risk: the drug is not included in any of the CredibleMeds TdP risk categories

NC Not Classified: the drug was reviewed by CredibleMeds but the evidence available was not enough to assign it to any of the previous categories

N Risperidone

In silico drug trials for Risperidone were run by considering the active moiety of Risperidone and its 9-OH metabolite (Paliperidone), which also affects cardiac ion channels[13].

**IC_{50}(h) when including Paliperidone**	I_{Na}	I_{CaL}	I_{Kr}	I_{Ks}
1x EFTPCmax	55.46(1)	15.25(1)	0.046(1)	136.364(1)
3x EFTPCmax	49.08(1)	15.25(1)	0.049(1)	136.364(1)
10x EFTPCmax	41.43(1)	15.25(1)	0.05(1)	136.364(1)
30x EFTPCmax	34.29(1)	15.24(1)	0.048(1)	136.364(1)
100x EFTPCmax	26.84(1)	15.22(1)	0.039(1)	136.364(1)

Amiodarone

The $EFTPC_{max}$ for Amiodarone was computed by considering C_{max}=2.5 µg/ml[5] and protein binding 96%[6]

Pentamidine

I_{Kr} IC_{50}/h for Pentamidine were set based on its effect on hERG trafficking[4]

* The $EFTPC_{max}$ for these drugs was computed from their C_{max}[2] and protein binding[6]

1 Merck internal database

2 www.pharmapendium.com (Accessed November 30, 2018)

3 Crumb, W. J., Vicente, J., Johannesen, L. & Strauss, D. G. An evaluation of 30 clinical drugs against the comprehensive in vitro Proarrhythmia assay proposed ion channel panel. J. Pharmacol. Toxicol. Methods 81, 251–262 (2016).

4 Kuryshev, Y.A., Ficker, E., Wang, L., Hawryluk, P., Dennis, A.T., Wible, B.A., et al. Pentamidine-Induced Long QT Syndrome and Block of hERG Trafficking. J. Pharmacol. Exp. Ther. 312: 316–323 (2005).

5 Kramer, J. et al. MICE models: superior to the HERG model in predicting Torsade de Pointes. Sci. Rep. 3, 2100 (2013).

6 Wishart, D. S. et al. DrugBank: a comprehensive resource for in silico drug discovery and exploration. Nucleic Acids Res. 34, D668-672 (2006).

7 Passini, E., Britton, O.J., Lu, H.R., Rohrbacher, J., Hermans, A.N., Gallacher, D.J., et al. (2017). Human In Silico Drug Trials Demonstrate Higher Accuracy than Animal Models in Predicting Clinical Pro-Arrhythmic Cardiotoxicity. Front. Physiol. *8* : 1–15.

8 Woosley, R., and Romer, K. (1999). www.crediblemeds.org, QTdrugs List. AZCERT, Inc. 1822 Innov. Park Dr., Oro Val. AZ 85755. Available online at: www.crediblemeds.org (Accessed November 30, 2018).

9 Lacerda, A.E., Kuryshev, Y.A., Yan, G.X., Waldo, A.L., and Brown, A.M. (2010). Vanoxerine: Cellular mechanism of a new antiarrhythmic. J. Cardiovasc. Electrophysiol. 21: 301–310.
